# DSD-LI-PO: A Bioinspired Parrot Optimizer with Componentwise Swarm Hybridization and Contracted Lens Imaging Refinement for Constrained Engineering Optimization

**DOI:** 10.3390/biomimetics11080598

**Published:** 2026-08-20

**Authors:** Meiqi Qin, Yongmei Ding

**Affiliations:** School of Mathematics and Systems Science, Wuhan University of Science and Technology, Wuhan 430065, China; qinmeiqi@wust.edu.cn

**Keywords:** Parrot Optimizer, swarm intelligence, dynamic swarm dimension hybridization, lens imaging, constrained engineering optimization

## Abstract

Bioinspired swarm optimizers are widely used for constrained engineering optimization, yet whole-vector updates can disrupt well-evolved components and insufficient elite refinement can limit late-stage accuracy. This study proposes DSD-LI-PO, an enhanced Parrot Optimizer (PO) integrating dynamic swarm dimension hybridization (DSD) with stagnation-triggered variable precision lens imaging (LI). DSD selectively inherits components from the current best solution to preserve useful dimensional information, whereas LI generates a progressively contracted elite-derived candidate when stagnation occurs. DSD-LI-PO is evaluated on CEC2017, six representative functions at 30, 50, and 100 dimensions, ablation variants, and five constrained engineering design problems. On CEC2017, it ranks second overall with an average rank of 1.8966 and records 28/1/0 wins/ties/losses against PO and 29/0/0 against IPO. In the scalability tests, it remains second in all three dimensions, with average ranks of 1.9167, 1.9167, and 1.7500. The complete method ranks first in the ablation study and second in the engineering tests, with feasible solutions in all runs. These results demonstrate that DSD-LI-PO provides a consistent and targeted improvement over PO while maintaining competitive performance against strong non-PO optimizers.

## 1. Introduction

Constrained engineering optimization often involves nonlinear objectives, coupled variables, irregular feasible regions, and inequality constraints, which can make gradient-based methods difficult to apply when derivative information is unavailable or the search landscape is complex [1,2,3,4]. Bioinspired and evolutionary optimizers provide derivative-free alternatives by searching with populations of candidate solutions and have therefore been widely studied for numerical and engineering optimization [5,6,7]. Optimization is also increasingly embedded in simulation-based engineering workflows; for example, IRoSim integrates CAD-based robotic task planning with trajectory and time optimization [8]. Their performance, however, still depends strongly on how information is exchanged within the population and how promising solutions are refined.

The Parrot Optimizer (PO) is a recent swarm intelligence algorithm based on foraging, staying, communication, and fear responses [9]. Although recent studies have extended PO through different search mechanisms and applications, two issues remain relevant for single-objective numerical and constrained engineering optimization. First, the original behavioral rules mainly update solution vectors as a whole. When different variables evolve at different rates, these updates may disturb components that already contain useful information, especially on multimodal, hybrid, nonseparable, or constrained landscapes. Second, PO has no explicit mechanism that generates an additional elite-derived candidate when the global best solution stagnates. This may limit refinement accuracy in the later search stage.

To address these issues, DSD-LI-PO integrates two complementary mechanisms into the original PO framework. Dynamic swarm dimension hybridization (DSD) performs selective componentwise inheritance from the current best solution, allowing useful dimensional information to be exchanged without replacing the whole candidate vector. Variable precision lens imaging (LI) is activated after a predefined period of stagnation and generates a contracted candidate from the current best solution for greedy refinement and population injection. The proposed method therefore targets component disruption and stagnation separately while retaining the basic behavioral structure of PO. Detailed relationships with existing PO variants, componentwise search, and lens imaging methods are reviewed in Section 2.

The main contributions of this study are summarized as follows:

- A DSD strategy is introduced to perform variable-level information exchange within the PO framework and reduce unnecessary disturbance of well-evolved components caused by whole-vector updates.
- A stagnation-triggered LI strategy is developed to generate a contracted elite-derived candidate and provide an additional refinement opportunity when the current best solution stops improving.
- DSD-LI-PO is evaluated through the 2017 IEEE Congress on Evolutionary Computation (CEC2017) benchmark suite for single-objective bound-constrained real-parameter numerical optimization [10], dimensional scalability tests, ablation analysis, and five constrained engineering design problems. Average ranks, convergence metrics, runtime, Wilcoxon rank sum tests, and Cliff’s delta are used to assess accuracy, convergence behavior, computational cost, and statistical significance.

The remainder of this article is organized as follows. Section 2, Section 3 and Section 4 review related work, summarize the baseline PO, and present DSD-LI-PO. Section 5, Section 6 and Section 7 describe the experimental evaluation and constrained engineering applications, followed by the Discussion and Conclusions.

## 2. Related Work

### 2.1. Parrot Optimizer and Its Recent Variants

PO was introduced as a bioinspired swarm optimizer that models several behavioral patterns of parrots, including foraging, staying, communication, and fear responses [9]. Because it is recent and structurally simple, PO has attracted several follow-up extensions. For example, the multiobjective Parrot Optimizer extends PO by introducing external archive management and Pareto-dominance-based selection for multiobjective problems [11]. The chaotic Parrot Optimizer incorporates chaotic maps to strengthen diversification and reduce premature convergence [12]. SNCPO integrates competitive swarm learning and salp navigation into PO for global numerical optimization and extreme learning machine training [13]. IPO improves PO for global optimization and multilayer perceptron classification by modifying several behavioral search components [14]. Other recent variants modify PO for wireless sensor network node deployment or related applications by introducing new search phases, local restart components, or problem-specific adaptation mechanisms [15].

These studies show that PO is an active research topic, but their design emphases differ from the present work. Some variants focus on multiobjective archiving, chaotic initialization, competitive population partitioning, neural-network training, or domain-specific deployment. In contrast, DSD-LI-PO focuses on single-objective numerical and constrained engineering optimization and specifically targets two issues in the search behavior of the baseline PO: disturbance of useful components caused by whole-vector updates and insufficient elite refinement after stagnation. Table 1 summarizes the representative recent PO variants and their main distinctions from DSD-LI-PO.

### 2.2. Componentwise and Elite-Guided Search Mechanisms

Componentwise information exchange is widely used in evolutionary and swarm-based optimization. Differential evolution introduced mutation and crossover as variable-level recombination operations for global optimization [16]. Variable-interaction analysis and dimension-oriented inheritance further show that optimization can benefit from identifying, grouping, or selectively inheriting informative variables [17,18]. In swarm optimization, particle swarm optimization and dimension-selection mechanisms also reflect the importance of dimensionwise information preservation and update control [19,20]. Recent adaptive differential evolution studies provide further evidence that dynamic perturbation and adaptive strategy design can improve variable-level search behavior [21,22]. These studies suggest that variable-level operations can be useful when different dimensions exhibit different search requirements.

The DSD mechanism follows this general componentwise motivation, but it is designed within the PO behavioral update framework. Instead of introducing a full differential mutation operator or replacing the PO search rules, DSD uses a two-stage selection rule. An individual-level gate first determines whether hybridization is performed, and a dimension-level mask then decides which components inherit information from the current best solution. The decreasing probabilities reduce the inheritance frequency in the later stage, which helps limit unnecessary dependence on the current best solution.

### 2.3. Opposition-Based Learning and Lens Imaging Refinement

Opposition-based learning (OBL) generates complementary candidate solutions by considering opposite positions in the search space [23,24]. OBL and its adaptive variants have been used to improve initialization, population diversity, and search-space adaptation in different metaheuristic algorithms [25]. Lens imaging opposition-based learning further modifies the opposite-position idea by introducing a scaling factor so that the generated candidate can be dynamically adjusted [26].

The LI mechanism in this study differs from repeatedly applying full-range opposition during the entire search process. It is activated only when the global best solution has not improved for a predefined number of consecutive iterations. The generated candidate is a contracted lens imaging point controlled by a nonlinear scale factor, and it is evaluated through greedy selection before being injected into the population. Therefore, LI is used as a stagnation-triggered elite refinement mechanism rather than a general random diversification operator.

### 2.4. Metaheuristics for Constrained Engineering Optimization

Constrained engineering optimization remains a common application area for metaheuristic algorithms because many design models include nonlinear constraints, mixed variable types, and irregular feasible regions [1,3]. Classical constrained benchmarks such as pressure vessel design, welded beam design, tension/compression spring design, speed reducer design, and cantilever beam design are frequently used to compare algorithmic feasibility, robustness, and search accuracy under controlled settings.

However, these benchmark problems should be interpreted carefully. They provide standardized constrained test cases rather than direct evidence of industrial deployment. For this reason, the present study uses them to examine whether DSD-LI-PO can maintain feasible and competitive behavior under constrained benchmark settings, while the Discussion section explicitly distinguishes benchmark-level validation from large-scale simulation-based or physical engineering applications.

## 3. Baseline Parrot Optimizer

### 3.1. Basic Model of PO

PO [9] is a swarm intelligence algorithm inspired by several social behaviors of parrots. In each iteration, individuals are updated through one of four behavioral modes: foraging, staying, communication, and fear response. These behaviors provide different movement patterns for global exploration, local perturbation, information exchange, and escape from unfavorable regions.

Similar to other population-based metaheuristic algorithms, PO starts with a population of candidate solutions randomly generated within the given search boundaries:

(1) $$X_{i}^{0}=lb+r\left(ub-lb\right),\;r∼U\left(0,1\right),$$

where lb and ub are the lower and upper boundary vectors, respectively. The position of individual *i* at iteration *t* is denoted as

(2) $$X_{i}^{t}=\left(x_{i,1}^{t},x_{i,2}^{t},\ldots ,x_{i,D}^{t}\right),$$

where *D* is the problem dimension.

Table 2 summarizes the four behavioral modes instead of repeating all original PO update equations. This compressed presentation keeps the baseline mechanism clear while leaving the main methodological focus on the two modifications introduced in DSD-LI-PO.

### 3.2. Limitations of PO

Although PO uses four behavioral patterns to balance exploration and exploitation, its update rules are mainly implemented in a whole-vector manner. This design is simple and efficient, but it can be less suitable when different variables evolve at different rates or have different constraint effects. In multimodal, hybrid, nonseparable, or constrained landscapes, some dimensions of an individual may already contain useful information, whereas others may still require broader search. A whole-vector movement may therefore overwrite useful components when the entire individual is perturbed by poorly evolved dimensions or stochastic search terms [17,20,27].

A second limitation is the lack of an explicit elite-based candidate generation mechanism under stagnation. In the later search stage, PO mainly relies on its original behavioral updates to refine candidate solutions. These updates do not generate an additional contracted complementary candidate for the current best solution. As a result, the algorithm may converge slowly near promising regions or retain residual errors when higher convergence accuracy is required.

These limitations motivate the two modifications developed in Section 4: DSD introduces selective componentwise information exchange after the original PO update, whereas LI adds a stagnation-triggered candidate-generation step for the current best solution.

## 4. Designed DSD-LI-PO

### 4.1. Motivation and Overall Framework

**Figure 1:**
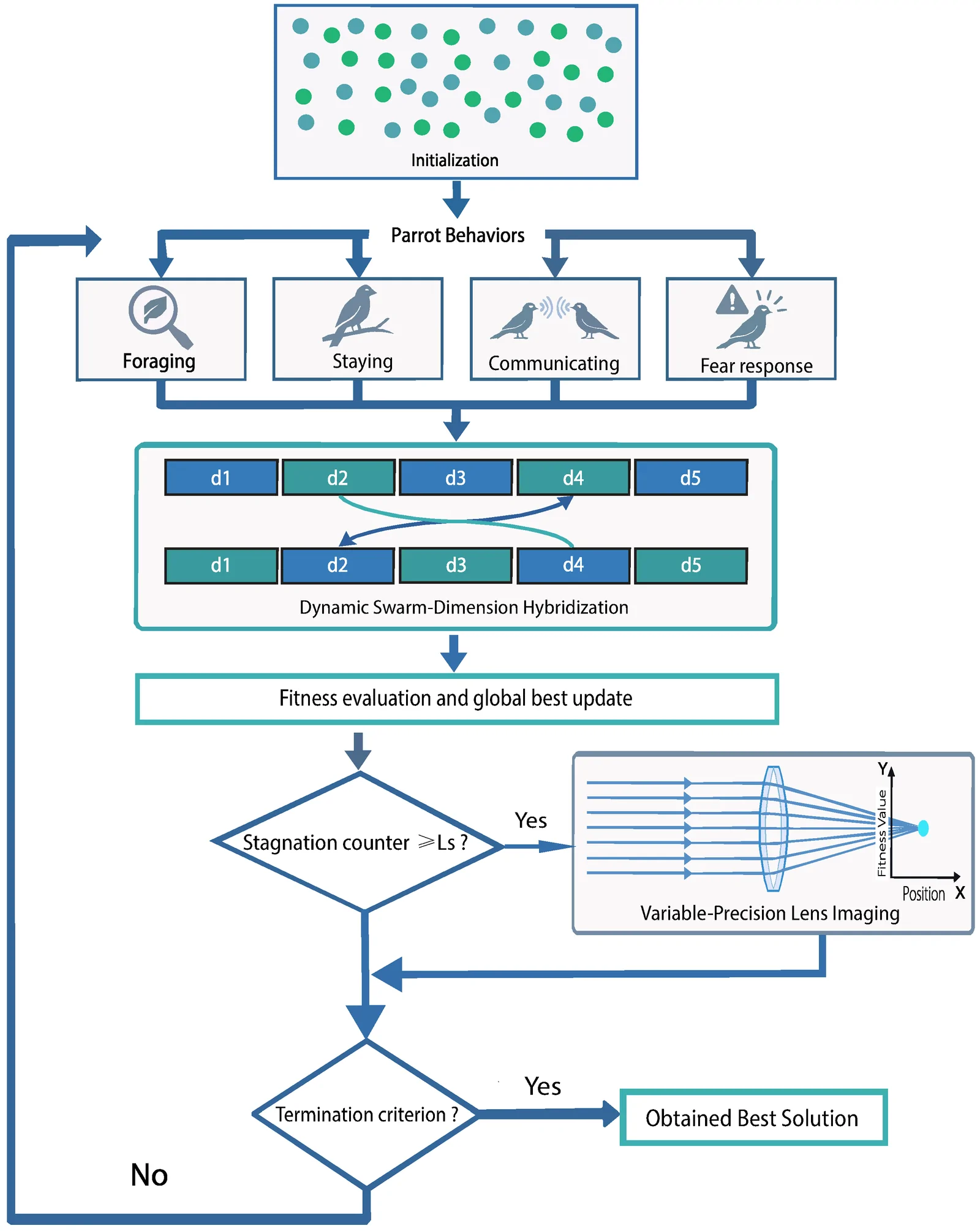
Overall framework of the designed DSD-LI-PO algorithm with componentwise hybridization and stagnation-triggered lens imaging refinement.

Based on the limitations above, DSD-LI-PO integrates two complementary mechanisms into the original PO framework. After each PO behavioral update, DSD applies a time-dependent individual gate and dimension mask so that only selected components inherit information from the current global best solution. LI acts separately: when the best solution has not improved for $L_{s}$ consecutive iterations, it generates a contracted lens imaging candidate, evaluates it greedily, and injects it into the population. Figure 1 shows the resulting workflow and the correspondence between the two identified limitations and their respective modifications.

### 4.2. Dynamic Swarm Dimension Hybridization Strategy

In high-dimensional optimization, different dimensions of the same individual may not evolve synchronously [17,28]. Some dimensions may already contain useful information, whereas others may still need further exploration. For whole vector update rules, the whole vector is modified simultaneously. Such a mechanism is simple, but it may also disturb well-evolved dimensions when the update direction is affected by poorly evolved dimensions. This problem becomes more evident on hybrid or nonseparable functions, where variable interaction makes the contribution of each dimension uneven.

To reduce this componentwise interference, DSD introduces a selective inheritance mechanism. Let ${\tilde{X}}_{i}^{t+1}$ denote the candidate solution obtained after the original PO behavioral update. DSD first uses an individual-level hybridization gate controlled by CR(t). If this gate is activated, a component mask is generated for the dimensions of the individual. The selected dimensions inherit the corresponding components from the current global best solution, whereas the remaining dimensions keep the values produced by the original PO update. Related componentwise inheritance and crossover ideas have also appeared in adaptive evolutionary search [18,20,21,22].

The individual-level hybridization gate is controlled by a linearly decreasing recombination probability:

(3) $$CR\left(t\right)=CR_{\max}-\left(CR_{\max}-CR_{\min}\right)\frac{t}{T_{\max}},$$

where $CR_{\max}$ and $CR_{\min}$ denote the initial and final recombination probabilities, respectively, and $T_{\max}$ is the maximum number of iterations. In this study, $CR_{\max}=0.4$ and $CR_{\min}=0.05$.

When the hybridization gate is activated, the component mask is controlled by a dimension selection probability:

(4) $$p_{d}\left(t\right)=0.2\left(1-\frac{t}{T_{\max}}\right).$$

Based on CR(t) and $p_{d}\left(t\right)$, the componentwise hybridization rule is defined as

(5) $$X_{i,j}^{t+1}=\left\{\begin{array}{cc} X_{best,j}^{t}, & \mathrm{if}\;r_{i}<CR\left(t\right)\;\mathrm{and}\;r_{i,j}<p_{d}\left(t\right),\\ {\tilde{X}}_{i,j}^{t+1}, & \mathrm{otherwise}, \end{array}\right.$$

where ${\tilde{X}}_{i,j}^{t+1}$ denotes dimension *j* of the candidate solution obtained after the original PO update, $X_{best,j}^{t}$ denotes dimension *j* of the current global best solution, and $r_{i}$ and $r_{i,j}$ are random numbers uniformly distributed in [0,1].

**Figure 2:**
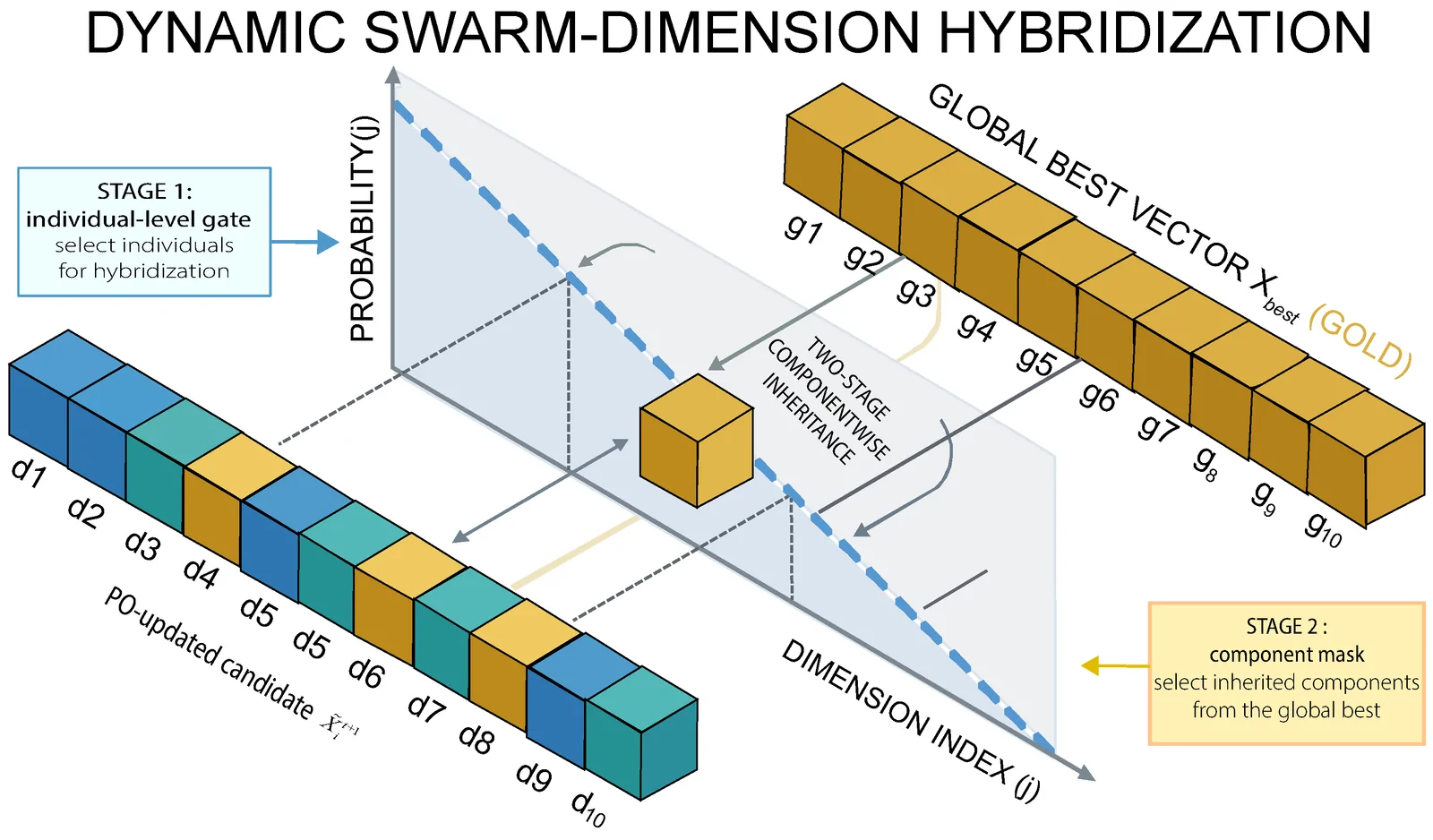
Two-stage componentwise information exchange in the DSD strategy.

The decreasing designs of CR(t) and $p_{d}\left(t\right)$ control how often information from the current global best solution is inherited. Early in the search, component exchange is more frequent, whereas both probabilities decrease as the search proceeds. This reduces explicit best-solution inheritance in later iterations and preserves the components generated by the original PO update. As illustrated in Figure 2, DSD does not replace the whole solution vector; it transfers selected components through a two-stage rule while leaving the remaining dimensions unchanged.

The amount of explicit best-solution inheritance can be quantified from Equations (3) and (4). For a particular component, the unconditional inheritance probability is

(6) $$P_{\mathrm{inh}}\left(t\right)=CR\left(t\right)p_{d}\left(t\right).$$

With the default settings, $P_{\mathrm{inh}}\left(0\right)=0.08$ and $P_{\mathrm{inh}}\left(0.5T_{\max}\right)=0.0225$, while it approaches zero as *t* approaches $T_{\max}$. For the main setting D=30, these probabilities correspond to expected inherited component counts of 2.4 at the beginning and 0.675 at the midpoint of the search, with the expected count approaching zero near the end. Thus, DSD uses the global best solution selectively rather than imposing persistent full-vector attraction, and its explicit dependence on best-solution inheritance decreases over time.

### 4.3. Variable Precision Lens Imaging

OBL [23,24] is often used to generate a complementary candidate solution from the opposite position of a given solution. However, the standard opposite solution is usually calculated with respect to fixed search boundaries. When the search process enters the later stage, directly using a full opposite solution may introduce an excessively large displacement and become less suitable for controlled elite solution updating [25].

To address this issue, this study introduces a stagnation-triggered variable precision lens imaging strategy. The proposed LI strategy generates a contracted lens imaging opposite candidate based on the current global best solution when the search process shows no improvement for several consecutive iterations. Let $L_{s}$ denote the stagnation threshold. In this study, $L_{s}=15$. When the current global best solution is not improved for $L_{s}$ consecutive iterations, LI is activated. Let $o_{j}$ be the center of dimension *j*, which is calculated as

(7) $$o_{j}=\frac{lb_{j}+ub_{j}}{2}.$$

The dynamic scaling factor is defined as

(8) $$k\left(t\right)=\left(1+{\left(\frac{t}{T_{\max}}\right)}^{\gamma }\right)\eta ,$$

where γ is the nonlinear adjustment coefficient and η is the baseline scaling factor. In this study, γ=0.5 and η=10. The lens imaging candidate of the current global best solution is calculated as

(9) $$X_{best,j}^{\prime }=o_{j}+\frac{o_{j}-X_{best,j}^{t}}{k(t)}=\frac{lb_{j}+ub_{j}}{2}+\frac{\frac{lb_{j}+ub_{j}}{2}-X_{best,j}^{t}}{k(t)},$$

where $X_{best,j}^{t}$ denotes dimension *j* of the current global best solution, and $lb_{j}$ and $ub_{j}$ are the lower and upper bounds of dimension *j*, respectively.

When k=1, Equation (9) degenerates into the standard opposite solution. In the implementation, k(t)>1, and therefore the generated candidate is a contracted opposite solution located between the search space center and the standard opposite point. As k(t) increases with the iteration process, the distance between the generated lens imaging candidate and the search space center decreases. Therefore, LI does not repeatedly perform full-range opposite search. Instead, it provides a dynamically contracted complementary candidate for the current global best solution [26].

Under the default setting γ=0.5 and η=10, k(t) increases from approximately 10 at the beginning of the search to 20 at the end. Accordingly, the offset of the LI candidate from the search-space center decreases from about 10% to 5% of the mirrored offset of the current best solution. This schedule makes LI a progressively more contracted complementary search step when stagnation occurs.

After the lens imaging candidate is generated, its fitness value is evaluated. If this candidate has a better fitness value than the current global best solution, it replaces the latter through greedy selection. Otherwise, the current global best solution is retained. In addition, the generated LI candidate is injected into the population by replacing the current worst individual. This operation allows the elite-derived complementary candidate to participate in the subsequent population update process.

**Figure 3:**
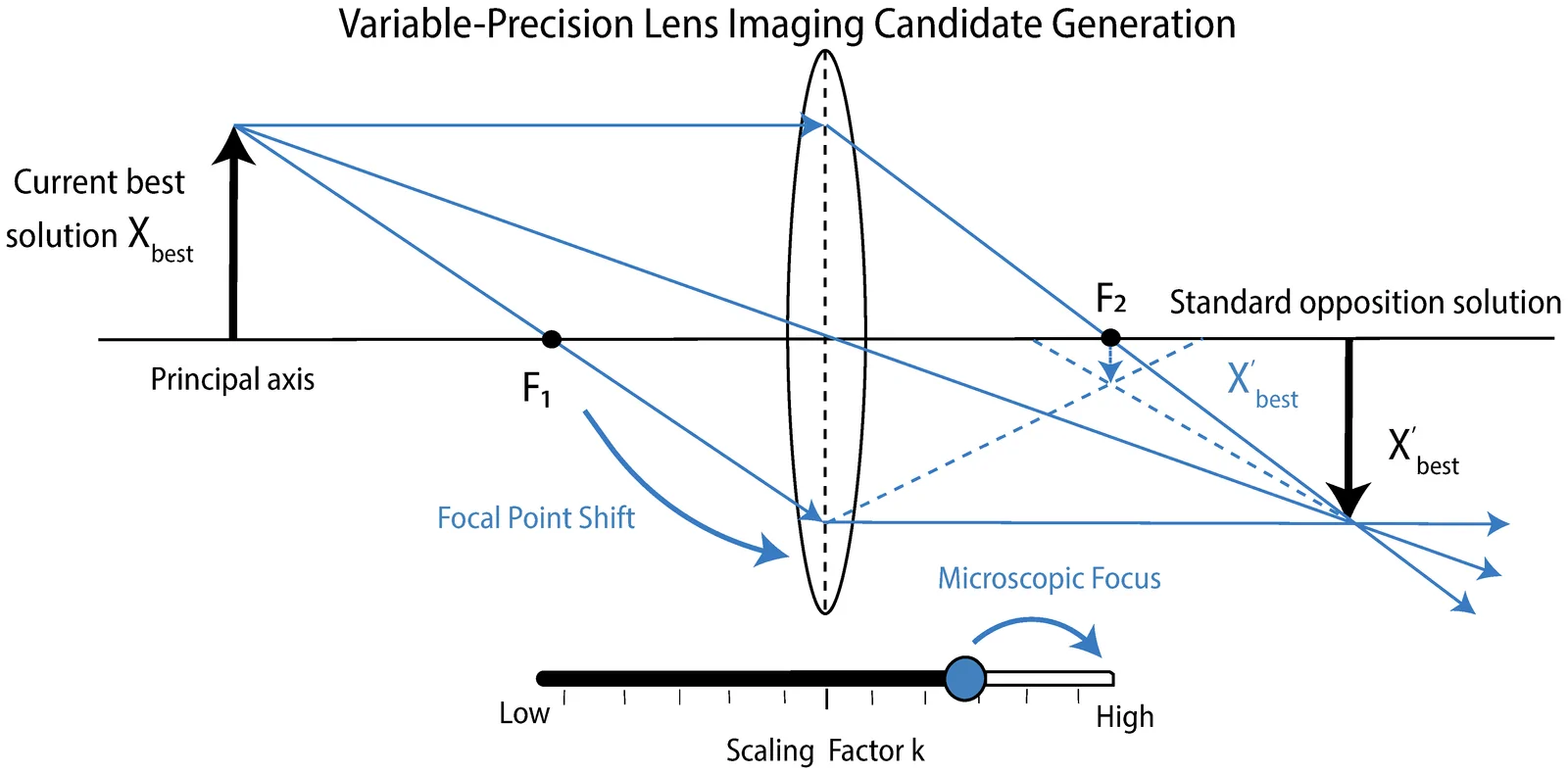
Geometric illustration of the contracted lens imaging candidate generation in LI.

Figure 3 illustrates the geometry of this operation. Because the candidate is contracted relative to standard opposition and generated only after stagnation, LI serves as an occasional complementary refinement step rather than a full-range opposition operator applied throughout the search.

### 4.4. Synergistic Mechanism and Algorithmic Procedure

DSD and LI operate at different stages of the search. After the original PO update, DSD selectively transfers components from the current best solution through the individual-level gate and dimension-level mask. LI is activated only after $L_{s}$ consecutive non-improving iterations and generates one contracted lens imaging candidate for additional elite refinement. Thus, DSD controls componentwise information exchange during routine population updating, whereas LI provides a stagnation-triggered refinement step. Algorithm 1 summarizes their integration in the complete DSD-LI-PO procedure.
**Algorithm 1** DSD-LI-PO**Require:** 
*f*, lb, ub, *N*, *D*, $T_{\max}$, and controls $\left(CR_{\max},CR_{\min},\gamma ,\eta ,L_{s}\right)$**Ensure:** 
Best solution $X_{best}$ and best fitness $f(X_{best})$1:Initialize population $X_{i}^{0}$, i=1,2,…,N, within [lb,ub]2:Evaluate all individuals and determine $X_{best}$3:Set stagnation counter $c_{s}\leftarrow 0$4:**for** t=1 to $T_{\max}$ **do**5:    **for** i=1 to *N* **do**6:        Generate ${\tilde{X}}_{i}^{t+1}$ using one original PO behavioral update rule7:        Compute CR(t) by Equation (3) and $p_{d}\left(t\right)$ by Equation (4)8:        **if** $r_{i}<CR\left(t\right)$ **then**9:           **for** j=1 to *D* **do**10:               **if** $r_{i,j}<p_{d}\left(t\right)$ **then**11:                   $X_{i,j}^{t+1}\leftarrow X_{best,j}^{t}$12:               **else**13:                   $X_{i,j}^{t+1}\leftarrow {\tilde{X}}_{i,j}^{t+1}$14:               **end if**15:           **end for**16:        **else**17:           $X_{i}^{t+1}\leftarrow {\tilde{X}}_{i}^{t+1}$18:        **end if**19:        Apply boundary correction to $X_{i}^{t+1}$ and evaluate fitness20:    **end for**21:    Update $X_{best}$ using the best individual in the population22:    **if** $X_{best}$ is improved **then**23:        $c_{s}\leftarrow 0$24:    **else**25:        $c_{s}\leftarrow c_{s}+1$26:    **end if**27:    **if** $c_{s}\geq L_{s}$ **then**28:        Generate the LI candidate $X_{best}^{\prime }$ using Equation (9)29:        Apply boundary correction and evaluate $X_{best}^{\prime }$30:        **if** $f\left(X_{best}^{\prime }\right)<f\left(X_{best}\right)$ **then**31:           $X_{best}\leftarrow X_{best}^{\prime }$32:        **end if**33:        Replace the worst individual in the population with $X_{best}^{\prime }$34:        $c_{s}\leftarrow 0$35:    **end if**36:**end for**37:**return** $X_{best}$ and $f(X_{best})$

### 4.5. Computational Complexity

The computational cost of DSD-LI-PO consists of population initialization, position updating, the DSD strategy, the stagnation-triggered LI strategy, and fitness evaluation. Let *N* denote the population size, *D* denote the problem dimension, $T_{\max}$ denote the maximum number of iterations, and $C_{f}$ denote the cost of one fitness evaluation. The initialization of the population requires O(ND) time, and the initial fitness evaluation requires $O(NC_{f})$ time.

In each iteration, the original PO behavioral update rules perform vector operations for all individuals and require O(ND) time. The DSD strategy performs individual-level gate checking, componentwise probability comparison, and assignment operations. Its time complexity is also O(ND) in each iteration. The LI strategy is activated only when the stagnation condition is satisfied. If LI is triggered, generating the contracted lens imaging candidate requires O(D) time, and evaluating this candidate introduces one additional fitness evaluation.

Let $N_{LI}$ denote the number of LI activations during the whole search process, where $0\leq N_{LI}\leq T_{\max}$. The total computational complexity can be expressed as

(10) $$O\left(ND+NC_{f}\right)+O\left(T_{\max}\left(ND+NC_{f}\right)\right)+O\left(N_{LI}\left(D+C_{f}\right)\right).$$

Since $N_{LI}\leq T_{\max}$ and the additional LI evaluation is applied to only one candidate when triggered, the dominant asymptotic order remains

(11) $$O\left(T_{\max}\left(ND+NC_{f}\right)\right).$$

When the fitness evaluation cost is proportional to the problem dimension, this reduces to $O(T_{\max}ND)$. Thus, DSD and LI add componentwise operations and occasional elite candidate evaluations, while keeping the same dominant asymptotic order as the original PO.

## 5. Experimental Design and Evaluation Protocol

This section describes the numerical evaluation protocol for DSD-LI-PO [29]. The evaluation comprises the main CEC2017 comparison, convergence analysis, dimensional scalability tests, parameter sensitivity analysis, and ablation experiments. The constrained engineering benchmarks are evaluated separately in Section 7.

### 5.1. Benchmark Functions and Compared Algorithms

The IEEE CEC2017 benchmark functions [10] are used to evaluate the numerical optimization performance of DSD-LI-PO. The benchmark functions provide different types of search landscapes, including unimodal, multimodal, hybrid, and composition characteristics, and are therefore suitable for examining convergence accuracy, local optimum avoidance, and robustness on complex search spaces.

DSD-LI-PO is compared with seven representative optimizers, including the original PO, improved Parrot Optimizer (IPO) [14], linear population size reduction success history based adaptive differential evolution (LSHADE) [30], Golden Jackal Optimization (GJO) [31], Dung Beetle Optimizer (DBO) [32], Whale Optimization Algorithm (WOA) [33], and Harris Hawks Optimization (HHO) [34]. The original PO is used as the direct baseline for evaluating the effect of the DSD and LI strategies. IPO is included as a recent PO variant to strengthen the comparison with PO-based methods. The IPO implementation follows the published mathematical formulation and algorithmic description in its original article. The other algorithms are selected as representative competitors with different search mechanisms, including differential evolution, swarm intelligence, and recently proposed population-based optimization methods.

All benchmark functions are treated as minimization problems. Final best fitness values are used for the main numerical comparison and ranking. For convergence visualization, the deviation from the best observed final fitness on each function is plotted with a logarithmic vertical axis, providing a common non-negative reference for the displayed curves. The same deviation sequence is used to derive the convergence-speed metrics.

### 5.2. Parameter Settings and Evaluation Criteria

For the main CEC2017 comparison, the population size was set to N=30, the problem dimension to D=30, and the maximum number of iterations to 1000. The search range was [−100,100], and each algorithm was independently executed 30 times on each function. Algorithm-specific parameters followed the original publications or commonly used settings.

All experiments were implemented under Linux 5.15.0 using Python 3.10.8, NumPy 1.24.1, and SciPy 1.15.3. Computations were performed on an AMD EPYC 9654 CPU-based AutoDL cloud instance with 32 vCPU cores and 60 GB memory.

Performance was summarized by the mean and standard deviation of the final best fitness values and by tie-aware average ranks. Pairwise differences between DSD-LI-PO and each comparator were evaluated with the Wilcoxon rank sum test [35,36] at the 0.05 significance level, together with Cliff’s delta as the effect-size measure [37]. The symbols “+”, “=”, and “−” denote significantly better, statistically similar, and significantly worse performance of DSD-LI-PO, respectively. Complete mean and standard deviation results are reported in Supplementary Table S1. Functionwise ranks, pairwise statistical tests, runtime summaries, detailed scalability results, sensitivity results, ablation results, and engineering validation statistics are provided in Supplementary Data S1.

## 6. Results

### 6.1. Overall Comparison on CEC2017

The expanded CEC2017 comparison results are summarized in Table 3, which reports the tie-aware average rank, overall rank, and average runtime per independent run.

Table 3 shows that HHO obtains the lowest average rank of 1.7586, followed closely by DSD-LI-PO with an average rank of 1.8966. DSD-LI-PO remains well ahead of the original PO (5.2414) and IPO (6.8966), which supports the effectiveness of the proposed modifications within the PO family. Its runtime is slightly higher than that of PO because of the componentwise operations and occasional LI evaluations, but it remains lower than the runtimes of IPO, GJO, and LSHADE under the same computing environment.

Complete functionwise mean ± standard deviation results for all compared algorithms are provided in Supplementary Table S1. These data complement the rank and statistical summaries with the absolute numerical performance on each CEC2017 function.

The pairwise statistical and effect-size summaries are reported in Table 4. This table complements the rank-based comparison by showing whether the observed differences are statistically reliable and by indicating their magnitude.

The statistical results confirm the main trend in Table 3. DSD-LI-PO is clearly better than IPO, DBO, and GJO in the pairwise summary, and it also shows strong advantages over PO and LSHADE. Compared with WOA, DSD-LI-PO shows only a limited number of significant wins, and most comparisons are statistically similar. Compared with HHO, DSD-LI-PO is mostly statistically similar and is worse in a small number of cases. These results support a cautious interpretation: DSD-LI-PO improves PO and performs strongly overall, but it does not uniformly dominate all competitors on every landscape type.

### 6.2. Results on Representative Benchmark Functions

**Figure 4:**
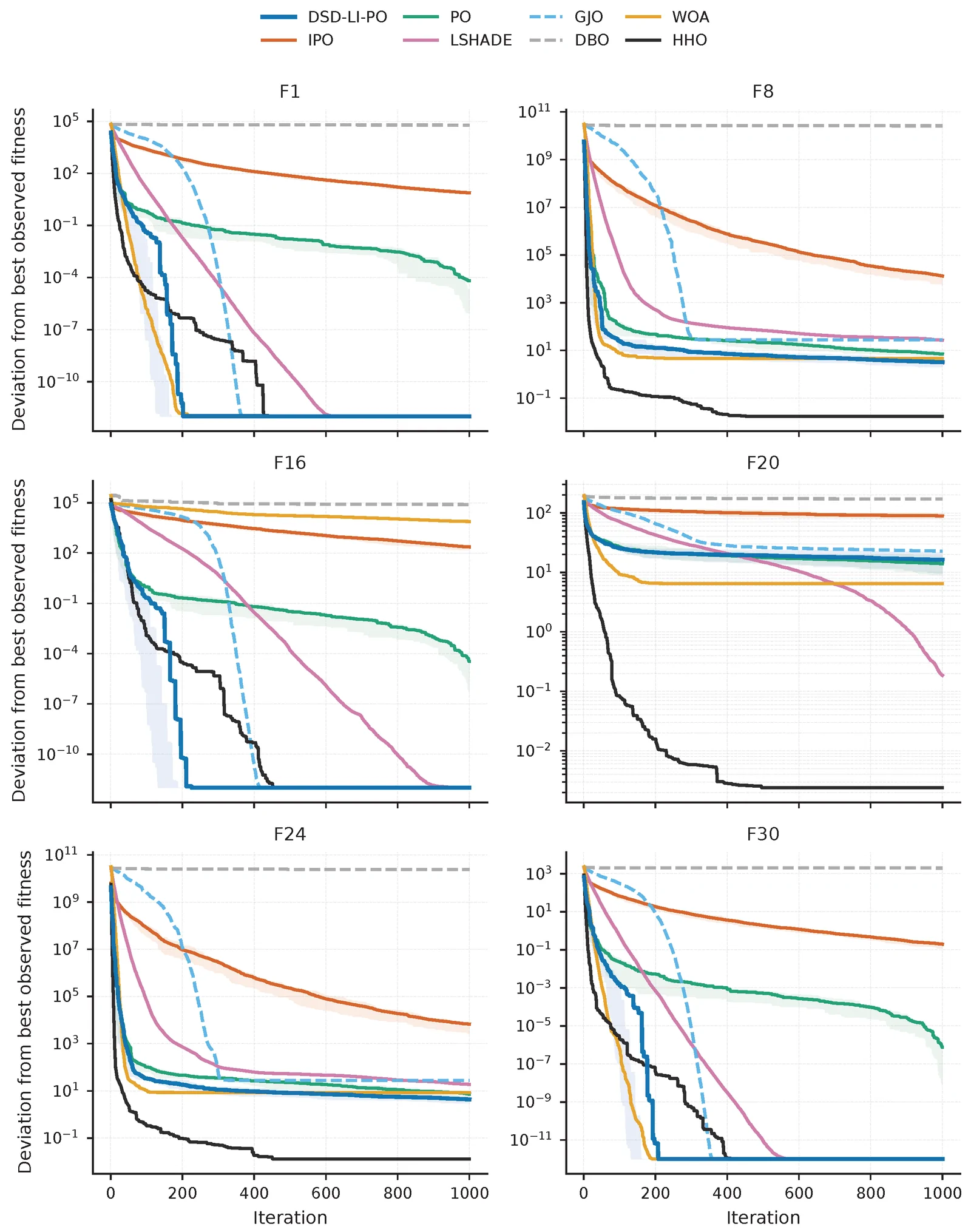
Convergence curves on six representative CEC2017 benchmark functions. Curves show the mean deviation from the best observed final fitness over 30 independent runs on a logarithmic vertical axis; the blue, orange, and green shaded bands correspond to DSD-LI-PO, IPO, and PO, respectively, and indicate the interquartile range (25th–75th percentiles).

To examine convergence behavior more directly, six representative CEC2017 functions are selected from different landscape types: F1, F8, F16, F20, F24, and F30. The convergence curves of all compared algorithms on these functions are shown in Figure 4.

Figure 4 shows that DSD-LI-PO reaches highly competitive final values on F1, F16, and F30, and remains among the leading algorithms on F8 and F24. HHO achieves the best final accuracy on F8, F20, and F24, which is consistent with the statistical summary in Table 4. The F20 result also shows that the proposed method is not uniformly optimal on all hybrid landscapes. Therefore, the convergence curves support the overall conclusion that DSD-LI-PO provides a strong and stable improvement over PO, while some problem landscapes still favor other search mechanisms.

A convergence-speed summary based on the same representative functions is provided in Table 5. The area under the logarithmic deviation sequence (AUC) reflects both convergence speed and residual deviation during the search process. Smaller values indicate faster reduction of the deviation from the best observed final fitness.

Table 5 indicates that HHO has the lowest AUC on the representative functions, while DSD-LI-PO obtains the second-lowest mean and median AUC values. This result is consistent with the convergence curves and prevents an overstatement that DSD-LI-PO is always the fastest converging method. A more accurate interpretation is that DSD-LI-PO provides competitive convergence speed while retaining the second-best overall average rank on the CEC2017 benchmark suite. The complete convergence curves of the main CEC2017 comparison are provided in Supplementary Figures S1–S3.

### 6.3. Scalability Analysis at Higher Dimensions

A separate dimensional scalability experiment was conducted on the six representative functions F1, F8, F16, F20, F24, and F30. The D=30 results were taken from the main CEC2017 experiment, while additional experiments were performed at D=50 and D=100. DSD-LI-PO was compared with PO, IPO, and HHO. For the additional experiments, the population size (N=30), maximum number of iterations (1000), search range ([−100,100]), and number of independent runs (30) were kept the same as in the main experiment. The corresponding dimensional scalability results are summarized in Table 6.

DSD-LI-PO ranks second among the four algorithms at all three dimensions, with average ranks of 1.9167, 1.9167, and 1.7500 at D=30, 50, and 100, respectively. Its pairwise statistical results are also unchanged across the three dimensions. DSD-LI-PO obtains 5/1/0 significant wins/ties/losses against PO and 6/0/0 against IPO at each dimension. Against HHO, the result is 0/3/3, showing statistical similarity on three functions and significantly worse performance on the other three.

The runtime of DSD-LI-PO increases from 0.9293 s at D=30 to 0.9423 s at D=50 and 1.0023 s at D=100. Together with the stable rank and pairwise results, these data show that the advantage of DSD-LI-PO over PO and IPO is maintained within the tested dimensional range. However, HHO retains the best average rank of 1.2500 at all three dimensions and remains significantly better on three of the six functions. The scalability results therefore support the stability of the proposed PO improvement from 30 to 100 dimensions rather than uniform superiority over all competing search mechanisms.

### 6.4. Sensitivity Analysis of Key Control Parameters

To further investigate the robustness of the parameter settings used in DSD-LI-PO, a small-scale sensitivity analysis was conducted on three key control components: the DSD probability schedule, the nonlinear scaling factor of LI, and the stagnation threshold used for LI activation. The default setting follows the parameter configuration adopted in the main experiments, while each sensitivity variant modifies one component around the default value. The analysis was performed on the same six representative CEC2017 functions using the same population size, dimension, and maximum iteration settings as the main comparison.

Table 7 shows that the tested configurations remain within a competitive performance range, while the DSD probability schedule has a more noticeable influence than the LI scaling factor. The DSD-Low setting achieves the lowest average rank and mean final deviation among the tested schedules, whereas DSD-High is less favorable on the representative functions. Changing γ produces comparatively small differences, and $L_{s}=10$ gives the lowest mean AUC among the tested stagnation thresholds. The sensitivity experiment is used as a diagnostic analysis rather than a post hoc tuning stage; therefore, the default configuration fixed for the main comparison is retained instead of being reselected from the representative-function results. The DSD schedule and stagnation threshold remain useful directions for future adaptive control.

### 6.5. Ablation Study

To examine the individual effects of the two strategies, the ablation study compares four variants: PO, DSD-PO, LI-PO, and DSD-LI-PO. PO denotes the original algorithm. DSD-PO introduces only the dynamic swarm dimension hybridization strategy. LI-PO introduces only the variable precision lens imaging strategy. DSD-LI-PO integrates both strategies.

Table 8 reports the overall ablation results of PO, DSD-PO, LI-PO, and DSD-LI-PO. The complete DSD-LI-PO obtains the best average rank of 1.5517 and the lowest mean and median absolute errors. LI-PO ranks second with an average rank of 1.5862, while DSD-PO improves the original PO from 3.5172 to 3.3448.

The separate variants confirm that both mechanisms contribute to the improvement over PO. LI-PO remains close to the complete method in average rank, while DSD-PO also improves the baseline. The combined DSD-LI-PO retains the best average rank and the lowest overall error indicators; the different roles of DSD and LI are discussed further in Section 8.

The +/=/− values in Table 8 are based on Wilcoxon rank sum tests using results from independent runs. Detailed functionwise ablation results and statistical tests are provided in Supplementary Data S1, and the complete ablation convergence curves are provided in Supplementary Figure S4.

## 7. Constrained Engineering Design Applications

To further examine the applicability of DSD-LI-PO to constrained engineering optimization tasks, five classical engineering design problems are used: pressure vessel design (PVD) [38], welded beam design (WBD) [31], tension/compression spring design (TCSD) [31], speed reducer design (SRD) [31], and cantilever beam design (CBD) [38]. These problems involve nonlinear objective functions, inequality constraints, and different types of design variables. They are commonly used as engineering benchmarks to evaluate the search performance of metaheuristic algorithms under constrained settings.

### 7.1. Experimental Setting for Engineering Problems

Table 9 shows that the selected engineering problems cover different constrained design settings. PVD and SRD include mixed variable characteristics, while WBD, TCSD, and CBD are continuous constrained problems with different dimensionalities and constraint numbers. This problem set is therefore suitable for examining whether DSD-LI-PO can maintain feasible and competitive search behavior beyond unconstrained benchmark functions. In the engineering experiments, DSD-LI-PO is compared with PO, LSHADE, WOA, and HHO. For all algorithms, the population size is set to 30, the maximum number of iterations is set to 500, and each algorithm is independently run 30 times on each engineering problem. The lower and upper bounds of the design variables follow the standard definitions used in the implemented engineering benchmark functions.

Penalty-function approaches are a widely used class of constraint-handling methods in evolutionary optimization [39,40,41]. In this study, a quadratic exterior penalty function with a fixed penalty coefficient is used to handle all inequality constraints. For a constrained problem with objective function f(x) and inequality constraints $g_{i}\left(x\right)\leq 0$, the penalized objective function is defined as

(12) $$F\left(x\right)=f\left(x\right)+\sum_{i}10^{15}\max{\left(0,g_{i}\left(x\right)\right)}^{2}.$$

Thus, feasible solutions keep their original objective values, whereas infeasible solutions receive a penalty proportional to the squared constraint violation. In the statistical comparison, the raw objective value is used for feasible runs, while the penalized fitness value is used for infeasible runs. The same penalized objective function and boundary settings are used for all compared algorithms. In PVD, the shell thickness and head thickness are discretized as multiples of 0.0625 by upward rounding before objective and constraint evaluation. In SRD, the number of teeth is treated as an integer variable. The complete mathematical formulations, including objective functions, constraints, and variable bounds, are provided in Supplementary Note S1.

### 7.2. Comparative Results

The statistical results of the compared algorithms on the five engineering design problems are summarized in Table 10.

Table 10 reports the best, mean, standard deviation, and worst values obtained over 30 independent runs. The results show that DSD-LI-PO obtains feasible and competitive solutions on the selected engineering design problems. According to the average rank based on the reported mean values in Table 10, where infeasible runs are evaluated using the penalized fitness value, LSHADE ranks first on this group of engineering benchmarks, while DSD-LI-PO ranks second. This ranking indicates that DSD-LI-PO improves the engineering performance of the original PO, although it does not dominate all compared algorithms.

Compared with the original PO, DSD-LI-PO reduces the reported mean value on PVD, TCSD, and SRD. The improvement is particularly clear on SRD, where the reported mean value is reduced from 3019.1771 to 3000.5087. The Wilcoxon rank sum test also shows that the improvement of DSD-LI-PO over PO on SRD is statistically significant at the 0.05 level. On PVD and TCSD, DSD-LI-PO obtains lower mean values than PO, but the differences are not statistically significant. On WBD and CBD, the two algorithms produce similar results, with PO giving slightly lower mean values.

DSD-LI-PO also performs better than WOA and HHO on most engineering problems. In particular, WOA and HHO suffer from large penalized fitness values in some constrained cases, such as TCSD and SRD, because infeasible runs are evaluated using the penalized objective function. In contrast, DSD-LI-PO obtains feasible solutions in all independent runs on the five engineering problems. These results indicate that the proposed strategies improve the engineering applicability of PO, although they do not make DSD-LI-PO uniformly superior to all competitors.

Table 11 lists the best feasible design variables obtained by DSD-LI-PO. These solutions correspond to the best objective values reported for DSD-LI-PO in Table 10 and provide the feasible design vectors identified by the algorithm.

Table 11 shows that DSD-LI-PO can generate feasible design solutions for all five engineering problems under the same experimental setting. For PVD and SRD, the reported variables are the repaired design variables used in objective and constraint evaluation, which is consistent with the mixed variable treatment described in Section 7.1. Additional engineering validation statistics are provided in Supplementary Data S1.

## 8. Discussion

The expanded CEC2017 comparison provides a broader view of the proposed method because it includes both the original PO and the recent IPO variant. With average ranks assigned to exact ties, DSD-LI-PO ranks second overall and retains a clear statistical advantage over PO and IPO. HHO obtains the lowest average rank and remains stronger on several representative functions. The main contribution of DSD-LI-PO is therefore its consistent and statistically supported improvement of the PO search framework while remaining competitive with strong non-PO optimizers.

The dimensional tests show that DSD-LI-PO preserves its relative advantage over PO and IPO from 30 to 100 dimensions. HHO remains stronger on part of the representative set, so the evidence supports stable performance over the tested dimensional range rather than unrestricted large-scale superiority.

The ablation results clarify the different contributions of the two mechanisms. DSD-PO improves on the original PO, indicating that selective componentwise inheritance improves the population-level information-exchange pattern. LI-PO achieves an average rank close to that of the complete method, showing that stagnation-triggered contracted refinement contributes strongly to final accuracy. DSD-LI-PO nevertheless obtains the best average rank and the lowest overall error indicators among the four variants, supporting the complementary use of DSD for componentwise cooperation and LI for stagnation relief.

The engineering benchmarks provide complementary evidence under constrained search. DSD-LI-PO obtains feasible solutions in all independent runs and improves the original PO on several problems, with a statistically significant improvement on speed reducer design. LSHADE remains the strongest method on this benchmark set, indicating that the relative performance of DSD-LI-PO still depends on the constraint structure and landscape characteristics of the problem.

The method is most suitable for single-objective continuous or mixed-variable optimization problems in which the search process may suffer from premature component disruption and stagnation around elite solutions. Typical examples include multimodal, hybrid, nonseparable, or constrained benchmark problems where different variables contribute unevenly to the objective and constraints. By contrast, direct use of DSD-LI-PO for multiobjective, dynamic, discrete combinatorial, or large-scale simulation-based engineering optimization would require additional mechanisms, such as archive management, dynamic response strategies, discrete encoding, or problem-specific constraint handling.

The benchmark functions and engineering models are treated as black-box minimization problems under the same computational budget, boundary handling, and quadratic exterior penalty treatment, while DSD and LI use fixed control settings. The engineering tests are standardized benchmark studies rather than physical or large-scale simulation-based deployments. These choices support a controlled algorithmic comparison, while industrial deployment would require domain-specific modeling and validation. Future work will therefore consider adaptive parameter control, more advanced constraint handling, and extensions to multiobjective, dynamic, discrete, and larger-scale simulation-based engineering optimization.

## 9. Conclusions

This study presented DSD-LI-PO, an enhanced PO variant that combines componentwise DSD with stagnation-triggered LI refinement. DSD regulates the transfer of current-best information at the component level, while LI adds a contracted candidate only when the best solution stops improving.

On the CEC2017 benchmark suite, DSD-LI-PO ranks second overall with a tie-aware average rank of 1.8966 and records 28/1/0 and 29/0/0 wins/ties/losses against PO and IPO, respectively. Its relative position is maintained in the 30-, 50-, and 100-dimensional scalability tests. The complete method also ranks first in the ablation study and second in the engineering tests, with feasible solutions obtained in all runs. Overall, the results support DSD-LI-PO as a consistent improvement of the PO framework for single-objective continuous or mixed-variable problems where componentwise progress is uneven and stagnation can restrict later search. Future work will examine adaptive control, advanced constraint handling, and extensions to multiobjective, dynamic, discrete, and larger-scale engineering optimization.

## Figures and Tables

**Table 1:** Comparison of representative recent PO variants and the present method.

Method	Main Modification	Primary Focus	Distinction from DSD-LI-PO
Multi-objective PO [11]	External archive and Pareto-based selection	Multiobjective global and engineering optimization	Emphasizes Pareto management rather than componentwise inheritance or stagnation-triggered elite refinement
Chaotic PO [12]	Chaotic search mechanisms	Diversification for complex optimization and image segmentation	Strengthens search diversity through chaotic behavior
SNCPO [13]	Competitive swarm learning and salp navigation	Global numerical optimization and extreme learning machine training	Uses a hybrid population-learning structure rather than the DSD–LI mechanism
IPO [14]	Modified PO behavioral search components	Global optimization and multilayer perceptron classification	Provides a recent PO-based comparator with a different behavioral redesign
DSD-LI-PO	Componentwise DSD and stagnation-triggered LI	Single-objective numerical and constrained engineering optimization	Targets whole-vector component disturbance and stagnant elite refinement within the original PO framework

**Table 2:** Summary of the four behavioral modes in the baseline PO.

Behavior	Main Search Role	Search Implication
Foraging	Movement guided by the current best solution, stochastic perturbation, and population information	Supports exploration of promising regions but updates the solution vector as a whole
Staying	Local perturbation around the current search state and best-solution information	Provides local variation while retaining stochastic movement
Communication	Information exchange based on population mean or decaying random movement	Maintains swarm-level interaction but still uses whole-vector operations
Fear response	Escape-oriented movement related to the current best solution and time-varying terms	Helps reduce premature aggregation but may also disturb well-evolved components

**Table 3:** Overall ranking and computational cost on the CEC2017 benchmark suite.

Algorithm	Average Rank	Overall Rank	Runtime (s)
HHO	1.7586	1	0.5912 ± 0.0833
DSD-LI-PO	1.8966	2	0.9271 ± 0.0854
WOA	2.8448	3	0.7132 ± 0.0821
GJO	4.2414	4	1.8349 ± 0.1024
LSHADE	5.1552	5	2.4669 ± 0.1167
PO	5.2414	6	0.8877 ± 0.0859
IPO	6.8966	7	1.0836 ± 0.0910
DBO	7.9655	8	0.1509 ± 0.0163

Note: Average ranks are calculated from functionwise mean final fitness values with average ranks assigned to exact ties. Runtime is reported as mean ± standard deviation over independent runs.

**Table 4:** Pairwise statistical comparison and effect-size summary on the CEC2017 benchmark suite.

Compared Algorithm	DSD-LI-PO +/=/−	Mean Cliff’s δ	Median Cliff’s δ
DBO	29/0/0	−1.0000	−1.0000
GJO	29/0/0	−0.8470	−0.8667
HHO	0/26/3	0.1011	0.0000
IPO	29/0/0	−1.0000	−1.0000
LSHADE	28/0/1	−0.9173	−1.0000
PO	28/1/0	−0.9338	−1.0000
WOA	5/23/1	−0.0577	0.0000

Note: Cliff’s δ is computed using the DSD-LI-PO samples relative to the compared algorithm samples. Negative values therefore indicate that DSD-LI-PO tends to obtain smaller final fitness values.

**Table 5:** Convergence-speed summary on representative CEC2017 benchmark functions.

Algorithm	Mean AUC	Median AUC	Mean Final Deviation	Median Final Deviation
HHO	−6.2364	−6.1790	0.0054	0.0012
DSD-LI-PO	−4.7985	−4.8297	3.9698	1.6089
WOA	−3.4281	0.2102	1284.6700	5.5072
LSHADE	−2.4137	−1.6030	7.6996	0.0931
GJO	−2.3024	−2.4580	12.9539	11.3906
PO	−0.5695	−0.2869	4.7478	3.5733
IPO	3.1510	2.6470	3381.9877	161.5537
DBO	6.0043	4.8638	$8.383\times 10^{9}$	$7.051\times 10^{4}$

Note: AUC is computed from the logarithmic deviation sequence. Lower AUC and lower final deviation indicate better convergence behavior under this metric.

**Table 6:** Additional dimensional scalability results on six representative CEC2017 benchmark functions.

Dimension	Average Rank				DSD-LI-PO +/=/−			DSD-LI-PO Runtime (s)
	**DSD-LI-PO**	**PO**	**IPO**	**HHO**	**vs. PO**	**vs. IPO**	**vs. HHO**	
30	1.9167	2.8333	4.0000	1.2500	5/1/0	6/0/0	0/3/3	0.9293
50	1.9167	2.8333	4.0000	1.2500	5/1/0	6/0/0	0/3/3	0.9423
100	1.7500	3.0000	4.0000	1.2500	5/1/0	6/0/0	0/3/3	1.0023

Note: Average ranks are recalculated among the four algorithms on the six representative functions, with average ranks assigned to exact ties. The +/=/− entries denote the numbers of functions on which DSD-LI-PO is significantly better than, statistically similar to, or significantly worse than the corresponding algorithm according to Wilcoxon rank sum tests at the 0.05 significance level. Runtime is the mean runtime per independent run averaged over the six functions.

**Table 7:** Sensitivity analysis of key control parameters on representative CEC2017 benchmark functions.

Group	Variant	Changed Setting	Avg. Rank	Mean AUC	Mean Final dev.	Runtime (s)
Baseline	Default	$CR_{\max}=0.4$, $CR_{\min}=0.05$, $p_{d,0}=0.2$, γ=0.5, $L_{s}=15$	2.3333	−4.8107	3.6618	0.9530
DSD schedule	DSD-Low	$CR_{\max}=0.3$, $CR_{\min}=0.03$, $p_{d,0}=0.15$	1.1667	−4.8674	2.9498	0.9310
DSD schedule	DSD-High	$CR_{\max}=0.5$, $CR_{\min}=0.10$, $p_{d,0}=0.25$	3.1667	−4.7836	4.2024	0.9337
LI scaling	K-Fast	γ=1.0, η=10	2.5000	−4.7940	3.8731	0.9277
LI scaling	K-Slow	γ=0.3, η=10	3.0000	−4.7780	3.9391	0.9247
Stagnation threshold	LS-Short	$L_{s}=10$	2.1667	−5.0725	3.4386	0.9272
Stagnation threshold	LS-Long	$L_{s}=20$	3.1667	−4.5588	3.7574	0.9261

Note: The sensitivity analysis was conducted on F1, F8, F16, F20, F24, and F30 with 10 independent runs for each setting. The default setting is the parameter configuration used in the main experiments. $p_{d,0}$ denotes the initial coefficient of the DSD dimension selection probability. AUC is computed from the logarithmic deviation sequence. Lower average rank, AUC, and final deviation indicate better performance under the corresponding metric. Full functionwise sensitivity results are provided in Supplementary Data S1. Avg. denotes the average value, and dev. denotes the standard deviation.

**Table 8:** Summary of ablation results on the considered CEC2017 benchmark functions.

Algorithm	Avg Rank	Mean Absolute Error	Median Absolute Error	+/=/−
DSD-LI-PO	1.5517	0.6099	0.5011	–
LI-PO	1.5862	0.7883	0.8421	1/28/0
DSD-PO	3.3448	0.7854	0.6892	28/1/0
PO	3.5172	0.9220	0.8475	28/1/0

Note: The +/=/− column reports the number of functions on which DSD-LI-PO is significantly better than, statistically similar to, or significantly worse than each compared variant, according to Wilcoxon rank sum tests using the results from 30 independent runs at the 0.05 significance level.

**Table 9:** Characteristics of the selected engineering design problems.

Problem	Variable Type	Dimension	Constraints	Main Characteristic
PVD	Mixed	4	4	Discretized thickness variables and nonlinear constraints
WBD	Continuous	4	7	Low dimensional constrained structural design
TCSD	Continuous	3	4	Low dimensional constrained mechanical design
SRD	Mixed	7	11	Integer teeth variable and multiple nonlinear constraints
CBD	Continuous	5	1	Continuous structural optimization problem

**Table 10:** Statistical results of the compared algorithms on engineering design problems.

Problem	Algorithm	Best	Mean	Std	Worst
PVD	DSD-LI-PO	5336.6226	5473.0385	125.8022	5789.9920
	PO	5371.8484	5531.3702	127.0481	5874.1060
	LSHADE	5336.6181	5345.6968	20.8797	5408.5057
	WOA	5870.5549	9074.7903	3723.3180	24,670.1100
	HHO	5777.8948	9148.7288	2431.9700	15,191.5200
WBD	DSD-LI-PO	1.7339	1.8231	0.1408	2.4526
	PO	1.7287	1.8073	0.0881	2.2287
	LSHADE	1.7249	1.7249	$3.34\times 10^{-16}$	1.7249
	WOA	1.9307	3.1196	0.9830	5.7360
	HHO	1.7972	2.8570	0.7939	4.6367
TCSD	DSD-LI-PO	0.012728	0.013713	0.001151	0.017748
	PO	0.012766	0.013923	0.001684	0.017791
	LSHADE	0.012665	0.012666	$1.18\times 10^{-6}$	0.012671
	WOA	0.012667	0.014012	0.001238	0.017805
	HHO	0.012785	$1.336609\times 10^{14}$	$1.336609\times 10^{14}$	$8.655466\times 10^{14}$
SRD	DSD-LI-PO	2986.5950	3000.5087	10.4477	3026.6048
	PO	2993.2570	3019.1771	25.1772	3101.2347
	LSHADE	2985.8609	2985.8609	$1.02\times 10^{-11}$	2985.8609
	WOA	3102.9708	$1.473756\times 10^{10}$	$7.936417\times 10^{10}$	$4.421267\times 10^{11}$
	HHO	3609.2332	$4.377840\times 10^{11}$	$7.084801\times 10^{11}$	$3.279169\times 10^{12}$
CBD	DSD-LI-PO	1.340406	1.343453	0.002843	1.354895
	PO	1.340745	1.343344	0.003210	1.354896
	LSHADE	1.339956	1.339956	$2.72\times 10^{-16}$	1.339956
	WOA	1.796195	2.745464	0.717418	4.398584
	HHO	1.378011	1.591333	0.137923	2.026176

Note: Best, mean, standard deviation, and worst are computed from the comparison values. For feasible runs, the comparison value is the raw objective value; for infeasible runs, it is the penalized fitness value.

**Table 11:** Best feasible design variables obtained by DSD-LI-PO on engineering design problems.

Problem	Best Objective Value	Design Variables
PVD	5336.622640	1.3125, 0.6250, 65.225262, 10.000000
WBD	1.733918	0.203491, 3.541172, 9.038247, 0.206089
TCSD	0.012728	0.050000, 0.317325, 14.043704
SRD	2986.594954	3.500031, 0.700000, 17.000000, 7.300000, 7.722029, 3.351401, 5.287087
CBD	1.340406	6.019353, 5.250677, 4.479897, 3.501562, 2.229377

## Data Availability

The numerical summaries, statistical results, dimensional scalability data, parameter sensitivity results, ablation results, engineering validation statistics, and engineering model formulations used in this study are provided in the Supplementary Materials and Supplementary Data S1. A public GitHub repository (v1.0.0) for the source code, experiment scripts, and reproducibility files is available at https://github.com/Eira-QmeiQ/DSD-LI-PO (accessed on 21 July 2026).

## Supplementary Materials

The following supporting information can be downloaded at: https://www.mdpi.com/article/10.3390/biomimetics11080598/s1, Supplementary Table S1, complete functionwise mean and standard deviation results for the CEC2017 comparison; Supplementary Figures S1–S3, complete convergence curves for the main CEC2017 comparison; Supplementary Figure S4, complete convergence curves for the ablation study; Supplementary Note S1, complete mathematical formulations of the five constrained engineering design problems; Supplementary Data S1, an Excel workbook containing functionwise ranks, pairwise statistical tests, runtime summaries, detailed dimensional scalability results, parameter sensitivity results, ablation results, and engineering validation statistics.
