# Supplementary material for: DSD-LI-PO: A Bioinspired Parrot Optimizer with Componentwise Swarm Hybridization and Contracted Lens Imaging Refinement for Constrained Engineering Optimization
Source: Biomimetics (Basel). 2026 Aug 20;11(8):598. doi: 10.3390/biomimetics11080598 (PMC13510081; doi:10.3390/biomimetics11080598)
Supplement: Supplementary file 1 [file biomimetics-11-00598-s001.zip › Supplementary_Materials.pdf]

Supplementary Materials

DSD-LI-PO: A Bioinspired Parrot Optimizer with Componentwise Swarm Hybridization and Contracted Lens Imaging Refinement for Constrained Engineering Optimization

Meiqi Qin Yongmei Ding

Contents. This file contains Supplementary Table S1 with complete functionwise mean and standard deviation results for the CEC2017 comparison, Supplementary Figures S1–S4, and Supplementary Note S1 with the complete formulations of the five constrained engineering design problems. Supplementary Data S1 is provided as a separate Excel workbook containing functionwise ranks, pairwise statistical tests, runtime summaries, detailed dimensional scalability results, parameter sensitivity results, ablation results, and engineering validation statistics.

Supplementary Table. Detailed numerical results on the CEC2017 benchmark suite

Table S1 reports the mean and standard deviation of the final best fitness values obtained by all compared algorithms over 30 independent runs.

Table S1: Detailed numerical results on the CEC2017 benchmark suite.

Function	DSD-LI-PO	IPO	PO	LSHADE	GJO	DBO	WOA	HHO
F1	1.000000E+02<br>± 0.000000E+00	1.076054E+02<br>± 2.466054E+00	1.000001E+02<br>± 1.975195E-04	1.000000E+02<br>± 8.618577E-15	1.000000E+02<br>± 6.623560E-15	6.142609E+04<br>± 7.976406E+03	1.000000E+02<br>± 0.000000E+00	1.000000E+02<br>± 0.000000E+00
F3	3.000000E+02<br>± 0.000000E+00	1.789124E+04<br>± 5.220310E+03	3.000004E+02<br>± 7.864428E-04	3.212698E+02<br>± 5.129536E+01	3.000000E+02<br>± 1.556244E-13	1.094854E+06<br>± 2.288897E+06	7.761411E+04<br>± 2.829825E+04	3.000000E+02<br>± 0.000000E+00
F4	4.000000E+02<br>± 0.000000E+00	4.180185E+02<br>± 3.424305E+00	4.000016E+02<br>± 2.096184E-03	4.009229E+02<br>± 6.540775E-01	4.000000E+02<br>± 4.515509E-14	4.864023E+02<br>± 3.565729E+00	4.000000E+02<br>± 1.734455E-14	4.000000E+02<br>± 0.000000E+00
F5	5.000000E+02<br>± 0.000000E+00	8.539921E+02<br>± 6.563468E+01	5.000103E+02<br>± 2.050401E-02	5.005306E+02<br>± 1.933063E+00	5.000000E+02<br>± 1.965338E-14	5.943752E+04<br>± 9.591687E+03	5.000000E+02<br>± 0.000000E+00	5.000000E+02<br>± 0.000000E+00
F6	6.000000E+02<br>± 0.000000E+00	6.200000E+02<br>± 1.666231E-09	6.000024E+02<br>± 2.621922E-03	6.200000E+02<br>± 2.756743E-09	6.205322E+02<br>± 9.524579E-02	6.200000E+02<br>± 0.000000E+00	6.000000E+02<br>± 3.468910E-14	6.000000E+02<br>± 0.000000E+00
F7	7.000000E+02<br>± 0.000000E+00	7.003848E+02<br>± 1.085494E-01	7.000000E+02<br>± 1.323179E-06	7.000089E+02<br>± 1.144707E-02	7.000000E+02<br>± 5.572162E-14	7.155193E+02<br>± 2.092317E+00	7.000000E+02<br>± 0.000000E+00	7.000000E+02<br>± 0.000000E+00
F8	8.032177E+02<br>± 1.940057E+00	1.400304E+04<br>± 1.554587E+04	8.071466E+02<br>± 3.363161E+00	8.270617E+02<br>± 3.014013E+01	8.275551E+02<br>± 8.484345E-01	2.609161E+10<br>± 6.246714E+09	8.045520E+02<br>± 9.646588E+00	8.000172E+02<br>± 2.659145E-02
F9	9.000000E+02<br>± 0.000000E+00	1.042696E+03<br>± 4.025065E+01	9.000017E+02<br>± 1.673715E-03	9.000309E+02<br>± 3.446818E-02	9.000000E+02<br>± 1.221247E-04	1.430078E+03<br>± 4.617450E+01	9.000000E+02<br>± 2.884328E-14	9.000000E+02<br>± 2.788922E-05
F10	1.000000E+03<br>± 0.000000E+00	1.384464E+03<br>± 8.402842E+01	1.000006E+03<br>± 2.034223E-02	1.001393E+03<br>± 5.300965E+00	1.000000E+03<br>± 2.075628E-14	5.839124E+04<br>± 7.387279E+03	1.000000E+03<br>± 0.000000E+00	1.000000E+03<br>± 0.000000E+00
F11	1.100000E+03<br>± 0.000000E+00	1.117984E+03<br>± 4.160653E+00	1.100014E+03<br>± 1.332520E-02	1.100000E+03<br>± 1.725826E-10	1.100000E+03<br>± 1.022673E-13	6.089938E+04<br>± 9.200772E+03	1.100000E+03<br>± 4.151256E-14	1.100000E+03<br>± 0.000000E+00
F12	1.200000E+03<br>± 0.000000E+00	2.791730E+03<br>± 4.211621E+02	1.200003E+03<br>± 6.866167E-03	1.205537E+03<br>± 1.300516E+01	1.200000E+03<br>± 1.059770E-13	5.983747E+04<br>± 6.489210E+03	1.200000E+03<br>± 0.000000E+00	1.200000E+03<br>± 0.000000E+00
F13	1.300000E+03<br>± 0.000000E+00	1.309789E+03<br>± 4.576356E+00	1.300003E+03<br>± 4.191005E-03	1.300000E+03<br>± 2.487899E-04	1.300000E+03<br>± 2.040984E-13	6.071557E+04<br>± 7.715854E+03	1.300000E+03<br>± 7.861352E-14	1.300000E+03<br>± 0.000000E+00
F14	1.400000E+03<br>± 0.000000E+00	1.428896E+03<br>± 9.870040E+00	1.400000E+03<br>± 1.442759E-04	1.400000E+03<br>± 2.291958E-13	1.400000E+03<br>± 8.618577E-14	7.041575E+04<br>± 9.249670E+03	1.400000E+03<br>± 8.410601E-13	1.400000E+03<br>± 0.000000E+00
F15	1.500000E+03<br>± 0.000000E+00	2.736979E+03<br>± 2.593948E+02	1.501504E+03<br>± 1.605988E+00	1.500000E+03<br>± 2.935046E-09	1.500000E+03<br>± 1.184231E-13	1.673802E+05<br>± 1.584874E+04	1.500000E+03<br>± 4.151256E-14	1.500000E+03<br>± 0.000000E+00
F16	1.600000E+03<br>± 0.000000E+00	1.833885E+03<br>± 1.268025E+02	1.600000E+03<br>± 7.338080E-05	1.600000E+03<br>± 2.496711E-13	1.600000E+03<br>± 9.250427E-14	8.129808E+04<br>± 1.208539E+04	9.288602E+03<br>± 1.538333E+04	1.600000E+03<br>± 0.000000E+00
F17	1.700000E+03<br>± 0.000000E+00	2.068110E+03<br>± 8.889939E+01	1.700006E+03<br>± 1.849704E-02	1.700531E+03<br>± 1.786658E+00	1.700000E+03<br>± 7.861352E-14	6.192180E+04<br>± 9.806846E+03	1.700000E+03<br>± 0.000000E+00	1.700000E+03<br>± 0.000000E+00
F18	1.800000E+03<br>± 0.000000E+00	1.072403E+13<br>± 5.507928E+13	1.800000E+03<br>± 4.937213E-05	1.800000E+03<br>± 1.933330E-13	1.800000E+03<br>± 1.059770E-13	4.674998E+39<br>± 1.639352E+40	1.800000E+03<br>± 0.000000E+00	1.800000E+03<br>± 0.000000E+00
F19	1.900000E+03<br>± 0.000000E+00	2.717047E+03<br>± 3.006880E+02	1.900009E+03<br>± 2.269838E-02	1.900000E+03<br>± 1.616988E-13	1.900000E+03<br>± 1.059770E-13	2.902461E+08<br>± 6.697049E+07	1.900000E+03<br>± 0.000000E+00	1.900000E+03<br>± 0.000000E+00
F20	1.988503E+03<br>± 6.662368E+00	2.061466E+03<br>± 1.617657E+01	1.986274E+03<br>± 6.982771E+00	1.972430E+03<br>± 2.808510E-01	1.995025E+03<br>± 2.571320E+00	2.143982E+03<br>± 9.862555E+00	1.978706E+03<br>± 7.327684E+00	1.972246E+03<br>± 6.079514E-03
F21	2.100000E+03<br>± 0.000000E+00	2.106359E+03<br>± 2.776633E+00	2.100000E+03<br>± 5.402288E-05	2.100000E+03<br>± 3.461193E-13	2.100000E+03<br>± 2.119539E-13	5.943986E+04<br>± 7.176664E+03	2.100000E+03<br>± 0.000000E+00	2.100000E+03<br>± 0.000000E+00
F22	2.200000E+03<br>± 0.000000E+00	2.578508E+03<br>± 7.977335E+01	2.200012E+03<br>± 3.620126E-02	2.200166E+03<br>± 7.428753E-01	2.200000E+03<br>± 1.850085E-13	6.362954E+04<br>± 7.208336E+03	2.200000E+03<br>± 0.000000E+00	2.200000E+03<br>± 0.000000E+00
F23	2.300000E+03<br>± 0.000000E+00	2.300002E+03<br>± 7.423988E-04	2.300000E+03<br>± 4.599649E-08	2.300000E+03<br>± 3.266884E-13	2.300000E+03<br>± 2.119539E-13	2.314841E+03<br>± 1.746289E+00	2.300000E+03<br>± 0.000000E+00	2.300000E+03<br>± 0.000000E+00
F24	2.404341E+03<br>± 2.233543E+00	9.157980E+03<br>± 6.816853E+03	2.407310E+03<br>± 3.477505E+00	2.418950E+03<br>± 1.939266E+01	2.427387E+03<br>± 6.091565E-01	2.420751E+10<br>± 6.692893E+09	2.408404E+03<br>± 1.243788E+01	2.400013E+03<br>± 2.618343E-02
F25	2.500000E+03<br>± 0.000000E+00	2.513108E+03<br>± 3.422295E+00	2.500016E+03<br>± 1.892526E-02	2.500000E+03<br>± 2.097252E-10	2.500000E+03<br>± 2.291958E-13	3.547990E+03<br>± 7.963912E+01	2.500000E+03<br>± 0.000000E+00	2.500000E+03<br>± 0.000000E+00
F26	2.600000E+03<br>± 0.000000E+00	2.627619E+03<br>± 9.010286E+00	2.600000E+03<br>± 2.036808E-04	2.600000E+03<br>± 4.614924E-13	2.600000E+03<br>± 2.265883E-13	7.220080E+04<br>± 1.068222E+04	2.600000E+03<br>± 2.099030E-12	2.600000E+03<br>± 0.000000E+00
F27	2.700000E+03<br>± 0.000000E+00	2.723426E+03<br>± 3.761521E+00	2.700437E+03<br>± 2.946364E-01	2.700192E+03<br>± 7.009697E-01	2.700000E+03<br>± 3.310252E-13	2.859019E+03<br>± 7.483460E+00	2.700000E+03<br>± 1.956246E-13	2.700000E+03<br>± 0.000000E+00
F28	2.800000E+03<br>± 0.000000E+00	3.175225E+03<br>± 8.370229E+01	2.800006E+03<br>± 1.218136E-02	2.800332E+03<br>± 1.638703E+00	2.800185E+03<br>± 1.013695E+00	6.397416E+04<br>± 8.997440E+03	2.800000E+03<br>± 0.000000E+00	2.800000E+03<br>± 0.000000E+00
F29	2.900000E+03<br>± 0.000000E+00	1.733935E+19<br>± 9.497138E+19	2.900016E+03<br>± 1.657116E-02	2.900000E+03<br>± 1.404924E-09	2.900000E+03<br>± 2.202042E-13	3.106497E+38<br>± 5.535143E+38	2.900000E+03<br>± 0.000000E+00	2.900000E+03<br>± 0.000000E+00
F30	3.000000E+03<br>± 0.000000E+00	3.000198E+03<br>± 9.019009E-02	3.000000E+03<br>± 1.724213E-06	3.000000E+03<br>± 2.119539E-13	3.000000E+03<br>± 2.291958E-13	4.985744E+03<br>± 2.876990E+02	3.000000E+03<br>± 0.000000E+00	3.000000E+03<br>± 0.000000E+00

Note: Each cell reports the mean on the first line and the standard deviation on the second line for the final best fitness value over 30 independent runs. Lower values are better.

Supplementary Figures

Supplementary Figures S1–S3 present the complete convergence curves for the main CEC2017 comparison. Supplementary Figure S4 presents the complete convergence curves for the ablation study. The main-comparison curves include DSD-LI-PO and all compared algorithms, while the ablation curves compare PO, DSD-PO, LI-PO, and DSD-LI-PO over 30 independent runs.

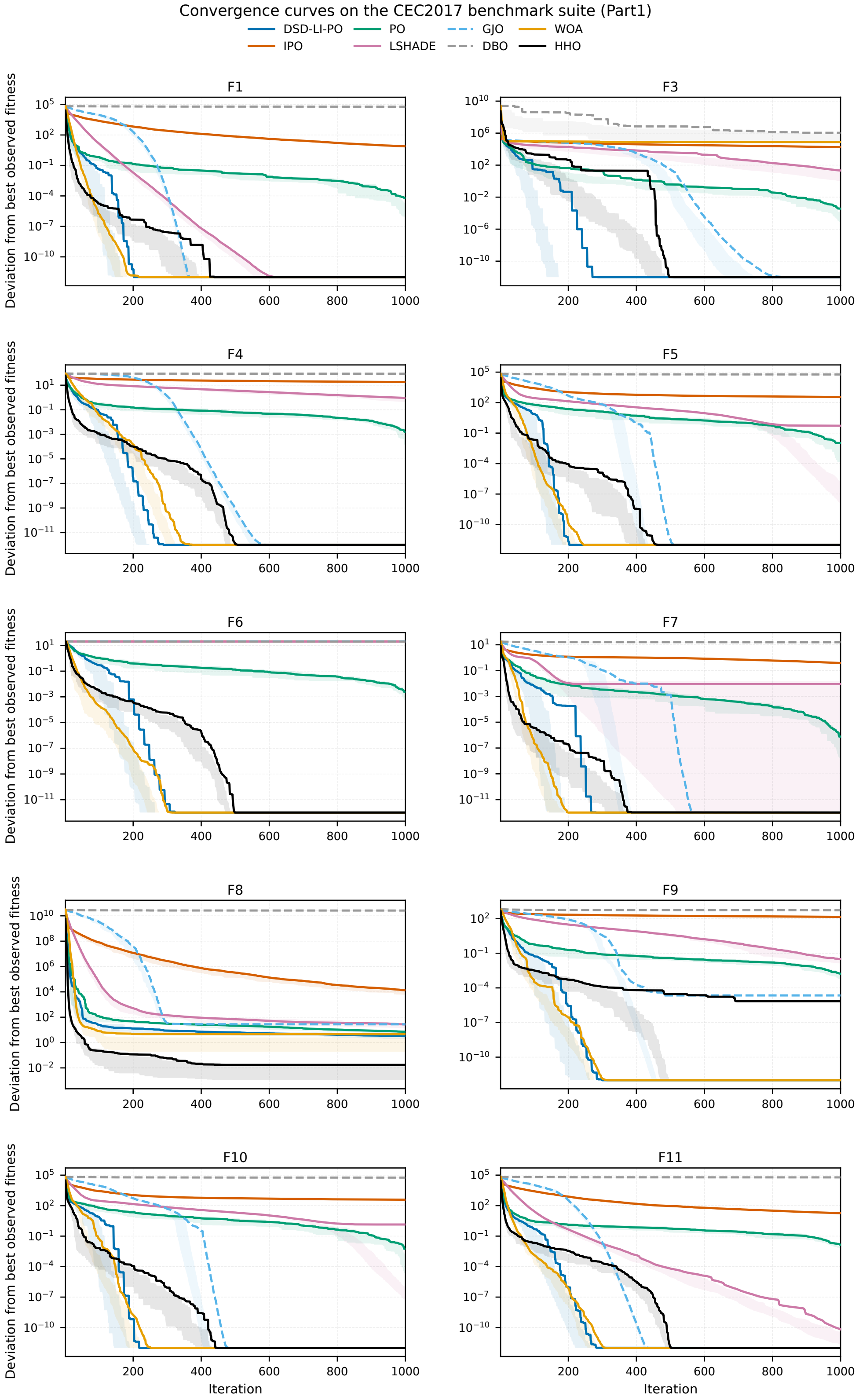

Figure S1: Complete convergence curves for the main CEC2017 comparison (Part 1).

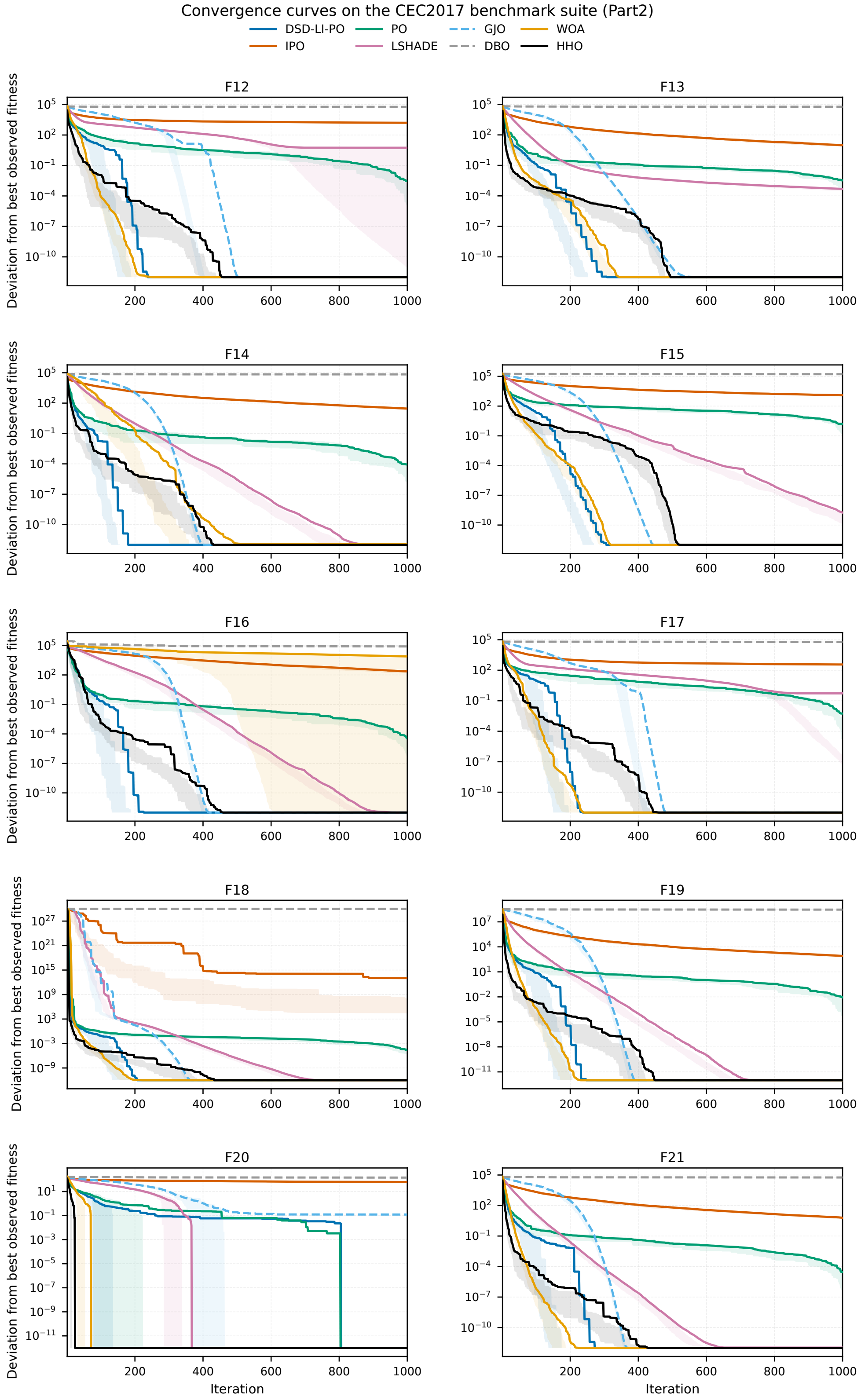

Figure S2: Complete convergence curves for the main CEC2017 comparison (Part 2).

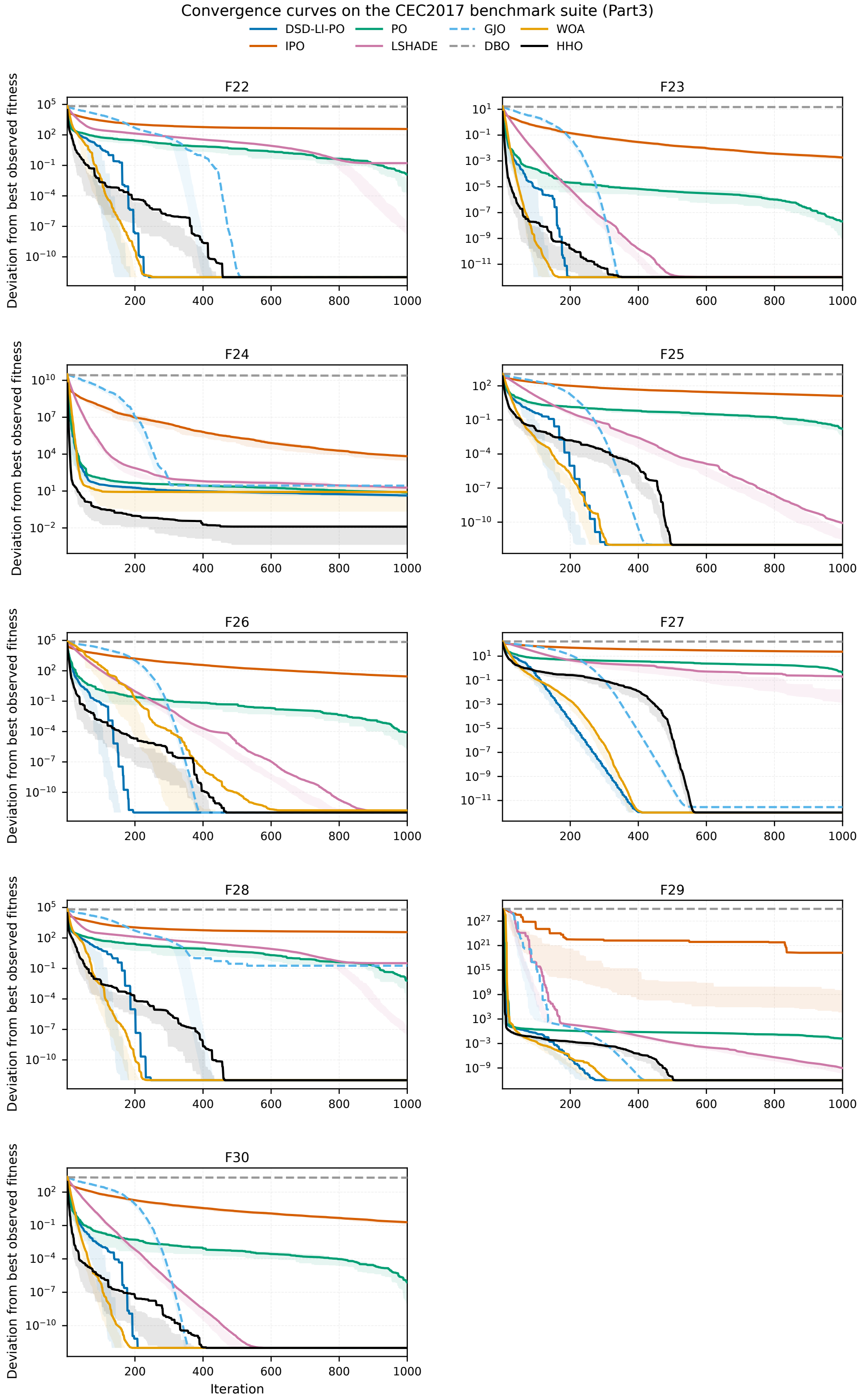

Figure S3: Complete convergence curves for the main CEC2017 comparison (Part 3).

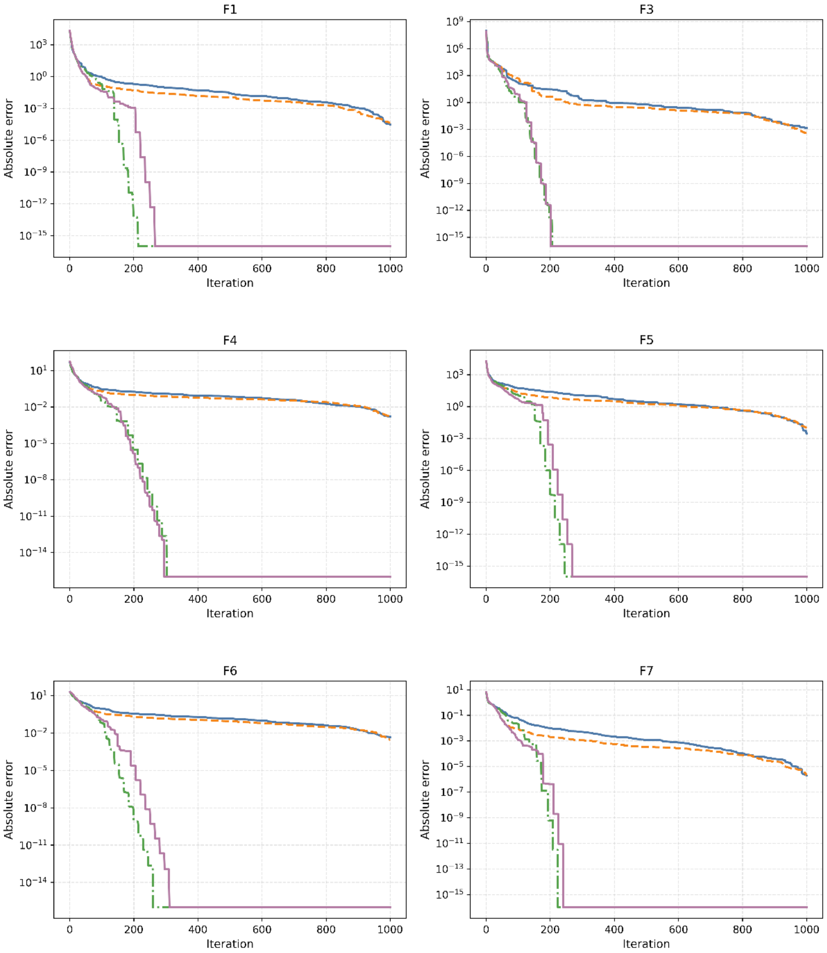

Figure S4: Complete convergence curves for the ablation experiment (Part 1 of 4).

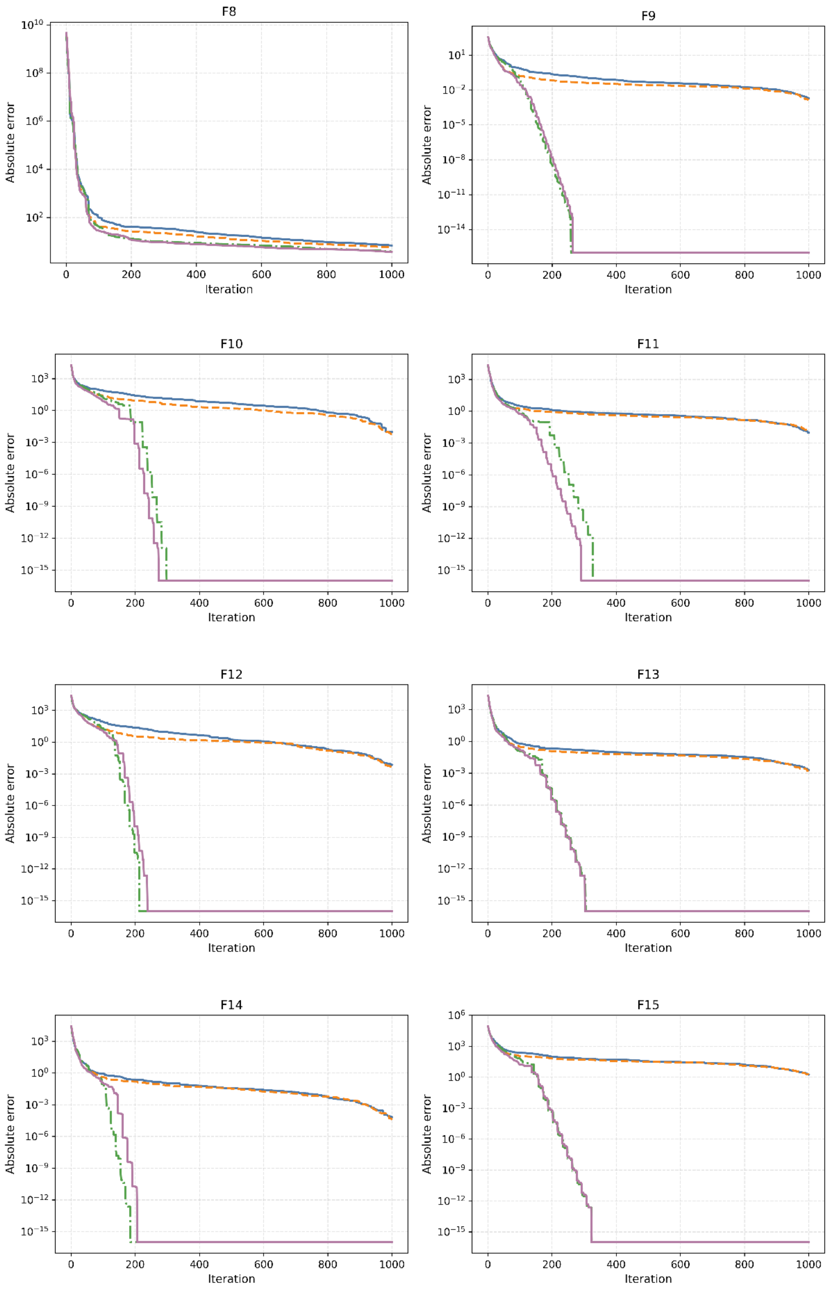

Figure S4: Complete convergence curves for the ablation experiment (Part 2 of 4).

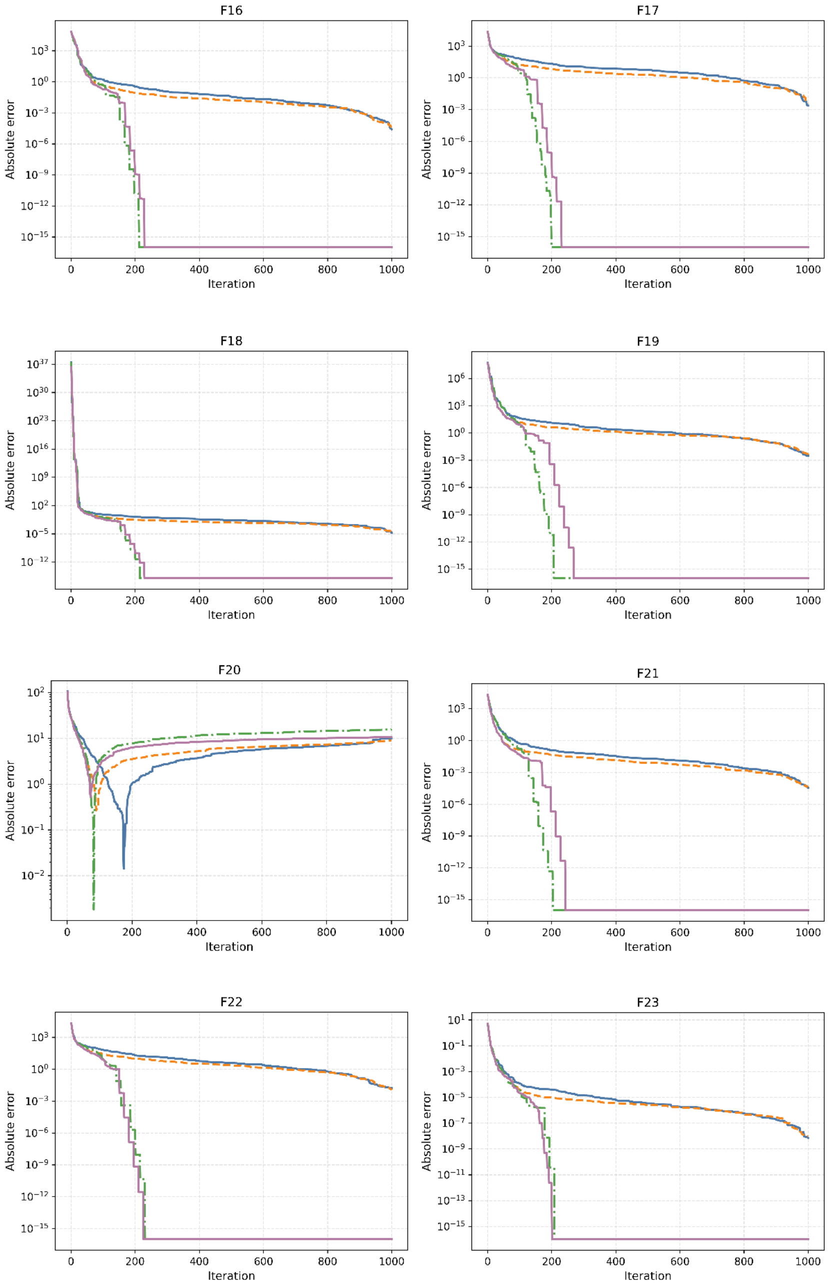

Figure S4: Complete convergence curves for the ablation experiment (Part 3 of 4).

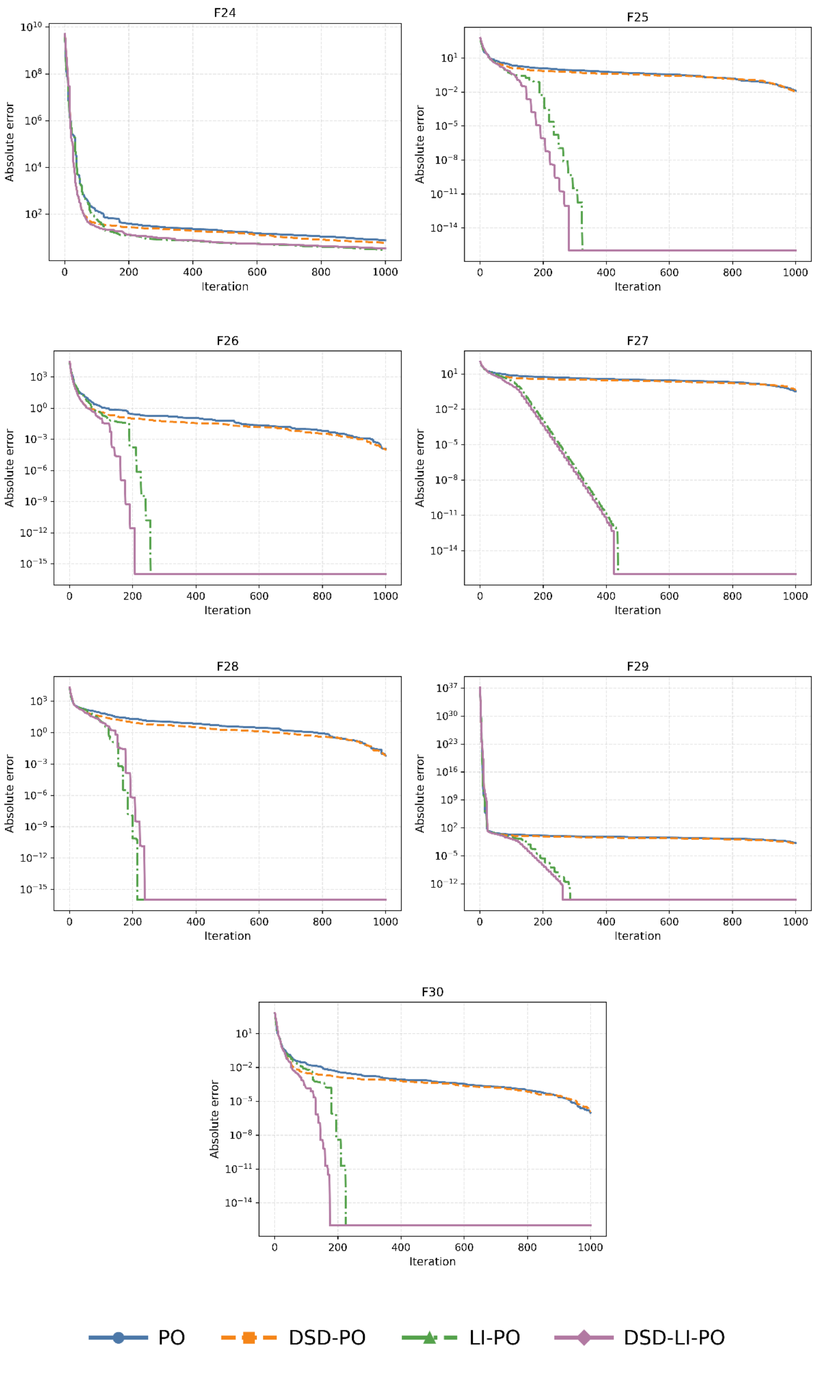

Figure S4: Complete convergence curves for the ablation experiment (Part 4 of 4).

Supplementary Data S1

The accompanying Excel workbook `Supplementary_Data_S1_final.xlsx` contains the numerical data used for the supplementary analyses. It includes the complete CEC2017 numerical summaries, tie-aware functionwise and overall ranks, pairwise Wilcoxon and Cliff's delta results, runtime summaries, detailed 30/50/100-dimensional scalability results, parameter sensitivity summaries and functionwise results, ablation results, and engineering validation statistics and best feasible designs.

Supplementary Note S1. Mathematical formulations of the constrained engineering design problems

This note provides the complete mathematical formulations of the five constrained engineering design problems used in the main manuscript. All problems are formulated as minimization problems with inequality constraints $g_i(x) \leq 0$.

0.1 Pressure vessel design problem

The pressure vessel design problem aims to minimize the total manufacturing cost of a cylindrical pressure vessel. The design vector is defined as

$$x = (x_1, x_2, x_3, x_4), \tag{S1}$$

where x_1 is the shell thickness, x_2 is the head thickness, x_3 is the inner radius, and x_4 is the cylindrical length. The objective function is

$$\min f(x) = 0.6224x_1x_3x_4 + 1.7781x_2x_3^2 + 3.1661x_1^2x_4 + 19.84x_1^2x_2. \tag{S2}$$

The constraints are

$$g_1(x) = -x_1 + 0.0193x_3 \leq 0, \tag{S3}$$
$$g_2(x) = -x_2 + 0.00954x_3 \leq 0, \tag{S4}$$
$$g_3(x) = -\pi x_3^2x_4 - \frac{4}{3}\pi x_3^3 + 1296000 \leq 0, \tag{S5}$$
$$g_4(x) = x_4 - 240 \leq 0. \tag{S6}$$

The variable bounds are

$$0.0625 \leq x_1 \leq 6.1875, \tag{S7}$$
$$0.0625 \leq x_2 \leq 6.1875, \tag{S8}$$
$$10 \leq x_3 \leq 200, \tag{S9}$$
$$10 \leq x_4 \leq 240. \tag{S10}$$

In this study, x_1 and x_2 are discretized as multiples of 0.0625 by upward rounding before objective and constraint evaluation.

0.2 Welded beam design problem

The welded beam design problem minimizes the fabrication cost of a welded beam. The design vector is defined as

$$x = (x_1, x_2, x_3, x_4), \tag{S11}$$

where x_1 is the weld thickness, x_2 is the weld length, x_3 is the beam height, and x_4 is the beam width. The objective function is

$$\min f(x) = 1.10471x_1^2x_2 + 0.04811x_3x_4(14 + x_2). \tag{S12}$$

The auxiliary terms are defined as

$$\tau' = \frac{6000}{\sqrt{2}x_1x_2}, \tag{S13}$$

$$M = 6000\left(14 + \frac{x_2}{2}\right), \tag{S14}$$

$$R = \sqrt{\frac{x_2^2}{4} + \frac{(x_1 + x_3)^2}{4}}, \tag{S15}$$

$$J = 2\sqrt{2}x_1x_2\left(\frac{x_2^2}{12} + \frac{(x_1 + x_3)^2}{4}\right), \tag{S16}$$

$$\tau'' = \frac{MR}{J}, \tag{S17}$$

$$\tau = \sqrt{(\tau')^2 + \frac{2\tau'\tau'' x_2}{2R} + (\tau'')^2}, \tag{S18}$$

$$\sigma = \frac{504000}{x_3^2 x_4}, \tag{S19}$$

$$\delta = \frac{65856000}{30 \times 10^6 x_3^3 x_4}, \tag{S20}$$

$$P_c = \frac{4.013(30 \times 10^6)\sqrt{x_3^2 x_4^6/36}}{196}\left(1 - \frac{x_3}{28}\sqrt{\frac{30 \times 10^6}{4(12 \times 10^6)}}\right). \tag{S21}$$

The constraints are

$$g_1(x) = \tau - 13600 \le 0, \tag{S22}$$

$$g_2(x) = \sigma - 30000 \le 0, \tag{S23}$$

$$g_3(x) = x_1 - x_4 \le 0, \tag{S24}$$

$$g_4(x) = 0.10471x_1^2 + 0.04811x_3x_4(14 + x_2) - 5 \le 0, \tag{S25}$$

$$g_5(x) = 0.125 - x_1 \le 0, \tag{S26}$$

$$g_6(x) = \delta - 0.25 \le 0, \tag{S27}$$

$$g_7(x) = 6000 - P_c \le 0. \tag{S28}$$

The variable bounds are

$$0.1 \le x_1 \le 2, \tag{S29}$$

$$0.1 \le x_2 \le 10, \tag{S30}$$

$$0.1 \le x_3 \le 10, \tag{S31}$$

$$0.1 \le x_4 \le 2. \tag{S32}$$

0.3 Tension/compression spring design problem

The tension/compression spring design problem minimizes the spring weight. The design vector is defined as

$$x = (x_1, x_2, x_3), \tag{S33}$$

where x_1 is the wire diameter, x_2 is the mean coil diameter, and x_3 is the number of active coils. The objective function is

$$\min f(x) = (x_3 + 2)x_2x_1^2. \tag{S34}$$

The constraints are

$$g_1(x) = 1 - \frac{x_2^3 x_3}{71785 x_1^4} \leq 0, \tag{S35}$$

$$g_2(x) = \frac{4x_2^2 - x_1 x_2}{12566(x_2 x_1^3 - x_1^4)} + \frac{1}{5108 x_1^2} - 1 \leq 0, \tag{S36}$$

$$g_3(x) = 1 - \frac{140.45 x_1}{x_2^2 x_3} \leq 0, \tag{S37}$$

$$g_4(x) = \frac{x_2 + x_1}{1.5} - 1 \leq 0. \tag{S38}$$

The variable bounds are

$$0.05 \leq x_1 \leq 2, \tag{S39}$$

$$0.25 \leq x_2 \leq 1.3, \tag{S40}$$

$$2 \leq x_3 \leq 15. \tag{S41}$$

0.4 Speed reducer design problem

The speed reducer design problem minimizes the reducer weight. The design vector is defined as

$$x = (x_1, x_2, x_3, x_4, x_5, x_6, x_7), \tag{S42}$$

where x_1 is the face width, x_2 is the module, x_3 is the number of teeth, x_4 and x_5 are the shaft lengths, and x_6 and x_7 are the shaft diameters. The objective function is

$$\begin{aligned} \min f(x) = {} & 0.7854 x_1 x_2^2 (3.3333 x_3^2 + 14.9334 x_3 - 43.0934) \\ & - 1.5708 x_1 (x_6^2 + x_7^2) + 7.4777 (x_6^3 + x_7^3) + 0.7854 (x_4 x_6^2 + x_5 x_7^2). \end{aligned} \tag{S43}$$

The constraints are

$$g_1(x) = \frac{27}{x_1 x_2^2 x_3} - 1 \leq 0, \tag{S44}$$

$$g_2(x) = \frac{397.5}{x_1 x_2^2 x_3^2} - 1 \leq 0, \tag{S45}$$

$$g_3(x) = \frac{1.93 x_4^3}{x_2 x_3 x_6^4} - 1 \leq 0, \tag{S46}$$

$$g_4(x) = \frac{1.93 x_5^3}{x_2 x_3 x_7^4} - 1 \leq 0, \tag{S47}$$

$$g_5(x) = \frac{\sqrt{\left(\frac{745 x_4}{x_2 x_3}\right)^2 + 16.9 \times 10^6}}{110 x_6^3} - 1 \leq 0, \tag{S48}$$

$$g_6(x) = \frac{\sqrt{\left(\frac{745 x_5}{x_2 x_3}\right)^2 + 157.5 \times 10^6}}{85 x_7^3} - 1 \leq 0, \tag{S49}$$

$$g_7(x) = \frac{x_2 x_3}{40} - 1 \leq 0, \tag{S50}$$

$$g_8(x) = \frac{5 x_2}{x_1} - 1 \leq 0, \tag{S51}$$

$$g_9(x) = \frac{x_1}{12 x_2} - 1 \leq 0, \tag{S52}$$

$$g_{10}(x) = \frac{1.5 x_6 + 1.9}{x_4} - 1 \leq 0, \tag{S53}$$

$$g_{11}(x) = \frac{1.1 x_7 + 1.9}{x_5} - 1 \leq 0. \tag{S54}$$

The variable bounds are

$$2.6 \leq x_1 \leq 3.6, \tag{S55}$$
$$0.7 \leq x_2 \leq 0.8, \tag{S56}$$
$$17 \leq x_3 \leq 28, \tag{S57}$$
$$7.3 \leq x_4 \leq 8.3, \tag{S58}$$
$$7.3 \leq x_5 \leq 8.3, \tag{S59}$$
$$2.9 \leq x_6 \leq 3.9, \tag{S60}$$
$$5.0 \leq x_7 \leq 5.5. \tag{S61}$$

In this study, x_3, the number of teeth, is treated as an integer variable and rounded to the nearest integer before objective and constraint evaluation.

0.5 Cantilever beam design problem

The cantilever beam design problem minimizes the weight of a cantilever beam. The design vector is defined as

$$x = (x_1, x_2, x_3, x_4, x_5). \tag{S62}$$

The objective function is

$$\min f(x) = 0.0624 \sum_{i=1}^{5} x_i. \tag{S63}$$

The constraint is

$$g_1(x) = \frac{61}{x_1^3} + \frac{37}{x_2^3} + \frac{19}{x_3^3} + \frac{7}{x_4^3} + \frac{1}{x_5^3} - 1 \leq 0. \tag{S64}$$

The variable bounds are

$$0.01 \leq x_i \leq 100, \quad i = 1, 2, \ldots, 5. \tag{S65}$$
